# Plasma Concentrations and Cancer-Associated Mutations in Cell-Free Circulating DNA of Treatment-Naive Follicular Lymphoma for Improved Non-Invasive Diagnosis and Prognosis

**DOI:** 10.3389/fonc.2022.870487

**Published:** 2022-06-16

**Authors:** Tevfik Hatipoğlu, Esra Esmeray Sönmez, Xiaozhou Hu, Hongling Yuan, Ayça Erşen Danyeli, Ahmet Şeyhanlı, Tuğba Önal-Süzek, Weiwei Zhang, Burcu Akman, Aybüke Olgun, Sermin Özkal, İnci Alacacıoğlu, Mehmet Ali Özcan, Hua You, Can Küçük

**Affiliations:** ^1^ İzmir Biomedicine and Genome Center, İzmir, Turkey; ^2^ İzmir International Biomedicine and Genome Institute, Dokuz Eylül University, İzmir, Turkey; ^3^ Department of Pathology, Faculty of Medicine, Dokuz Eylül University, İzmir, Turkey; ^4^ Department of Hematology, Faculty of Medicine, Dokuz Eylül University, İzmir, Turkey; ^5^ Department of Bioinformatics, Graduate School of Natural and Applied Sciences, Muğla Sıtkı Koçman University, Muğla, Turkey; ^6^ Department of Pathology and Microbiology, University of Nebraska Medical Center, Omaha, NE, United States; ^7^ Department of Oncology, Affiliated Cancer Hospital and Institute of Guangzhou Medical University, Guangzhou, China; ^8^ Department of Medical Biology, Faculty of Medicine, Dokuz Eylül University, İzmir, Turkey

**Keywords:** follicular lymphoma, liquid biopsy, cfDNA genotyping, targeted ultra-deep sequencing, non-invasive diagnosis, prognosis, targeted therapy, histological transformation

## Abstract

Follicular lymphoma (FL) is the second most frequent non-Hodgkin lymphoma accounting for 10-20% of all lymphomas in western countries. As a clinically heterogeneous cancer, FL occasionally undergoes histological transformation to more aggressive B cell lymphoma types that are associated with poor prognosis. Here we evaluated the potential of circulating cell-free DNA (cfDNA) to improve the diagnosis and prognosis of follicular lymphoma patients. Twenty well-characterized FL cases (13 symptomatic and 7 asymptomatic) were prospectively included in this study. Plasma cfDNA, formalin-fixed paraffin-embedded (FFPE) tumor tissue DNA, and patient-matched granulocyte genomic DNA samples were obtained from 20 treatment-naive FL cases. Ultra-deep targeted next-generation sequencing was performed with these DNA samples by using a custom-designed platform including exons and exon-intron boundaries of 110 FL related genes. Using a strict computational bioinformatics pipeline, we identified 91 somatic variants in 31 genes in treatment-naive FL cases. Selected variants were cross-validated by using PCR-Sanger sequencing. We observed higher concentrations of cfDNA and a higher overlap of somatic variants present both in cfDNA and tumor tissue DNA in symptomatic FL cases compared to asymptomatic ones. Variants known to be associated with FL pathogenesis such as STAT6 p.D419 or EZH2 p.Y646 were observed in patient-matched cfDNA and tumor tissue samples. Consistent with previous observations, high Ki-67 staining, elevated LDH levels, FDG PET/CT positivity were associated with poor survival. High plasma cfDNA concentrations or the presence of *BCL2* mutations in cfDNA showed significant association with poor survival in treatment-naive patients. *BCL2* mutation evaluations in cfDNA improved the prognostic utility of previously established variables. In addition, we observed that a FL patient who had progressive disease contained histological transformation-associated gene (i.e. *B2M* and *BTG1*) mutations only in cfDNA. Pre-treatment concentrations and genotype of plasma cfDNA may be used as a liquid biopsy to improve diagnosis, risk stratification, and prediction of histological transformation. Targeted therapies related to oncogenic mutations may be applied based on cfDNA genotyping results. However, the results of this study need to be validated in a larger cohort of FL patients as the analyses conducted in this study have an exploratory nature.

## Introduction

Follicular lymphoma (FL) is the second most common type of non-Hodgkin lymphoma, which accounts for 20-30% of all non-Hodgkin lymphomas (1). FL originates from germinal center (GC) B cells (2), and displays substantial clinic and genetic heterogeneity (3). FL cases occasionally undergo histological transformation to more aggressive B cell lymphoma subtypes with poor prognosis such as diffuse large B cell lymphoma (DLBCL) or Burkitt lymphoma, which are usually associated with poor survival and chemotherapy resistance (4). Asymptomatic FL patients are usually observed without any chemotherapeutic intervention whereas symptomatic FL cases are treated with rituximab-containing immunochemotherapy regimens (R-CHOP, R-CVP, etc.) (5). In 85-90% of FL cases, the anti-apoptotic *BCL2* gene is overexpressed as a result of t(14, 18)(q32;q21) translocation (2).

Genetic aberrations other than *BCL2* translocations are required for FL development including somatic mutations of epigenetic regulatory genes (i.e. *CREBBP*, *EZH2*, and *KMT2D*) or the genes of the JAK-STAT pathway (e.g. *STAT6*, *SOCS1*) (6). *CREBBP* mutations were detected in *in situ* follicular neoplasia (7), a neoplasm recently reported to preceed follicular lymphoma (8). Hotspot mutations of *EZH2* (i.e.Y646, A682, and A692) were detected to be frequent and early event in FL pathogenesis (9). Among these *EZH2* mutations, the role of Y646 (previously referred as Y641) was reported to inhibit EZH2 activity *in vitro* in FL (10), thereby potentially reducing H3K27me3 histone marks, which is associated with transcriptional silencing. Importantly, genetic depletion of the histone lysine transferase KMT2D led to B cell lymphoma development *in vivo*, suggesting that it may act as a tumor suppressor of FL development (11).

Diagnostic and prognostic evaluations of follicular lymphoma require histological, immunohistochemical (e.g. CD19, CD20 staining), and molecular genetic analyses in tumor tissue sections of biopsies obtained through invasive procedures such as incisional biopsy (12). However, biopsies obtained through invasive methodologies have several disadvantages: 1) patients may feel some pain and have other complications related to the procedure; 2) tumor biopsy may be difficult to obtain, and repeated sampling is not feasible; 3) genetic landscape of the entire tumor tissue or tumors at metastatic sites cannot be fully identified; 4) monitoring response to immunochemotherapy treatment is challenging. Currently available non-invasive methods (e.g. PET, PET/CT) used for cancer diagnosis and staging are expensive (13). Moreover, they may generate false-positive results (14, 15); and the results of these imaging tests do not exclude the need for tumor tissue evaluation. To overcome these challenges associated with diagnosis and cancer patient management, circulating cell-free DNA (cfDNA) genotyping has been proposed as an alternative approach for diagnosis, prognostication, management, and monitoring of different cancers (16) including B cell lymphoma subtypes (6).

Plasma cfDNA derives from the genomic DNA of apoptotic or necrotic cells released into the blood circulation (17). In cancer patients, cfDNA includes circulating cell-free tumor DNA (ctDNA) fragments reflecting cancer genomes such that cancer-associated genetic alterations including point mutations can be detected in plasma cfDNA samples when appropriate methodologies are used. Indeed, this possibility was already shown for a variety of carcinoma types such as lung (18) or prostate cancer (19). Recent studies showed the feasibility of ctDNA genotyping for diagnosis, prognosis, and monitoring response to therapy of classical Hodgkin lymphoma (20) and diffuse large B-cell lymphoma (21). However, the clinical potential of cfDNA genotyping in follicular lymphoma has been largely unknown. In this study, our main goal was to address whether it is possible to identify cancer-associated or prognostically-related mutations in plasma cfDNA samples of FL patients which may provide basis for future investigations focusing on non-invasive diagnosis and risk stratification of patients for improved patient management.

## Materials and Methods

### FL Patients and Clinicopathological Data

Twenty FL cases diagnosed and clinically followed up in Dokuz Eylül University hospital were included in this study. The patient samples used in this study were approved by the institutional review board of the Medical School at Dokuz Eylül University (protocol no: 2233-GOA). All patients consent to participate in the study. All FL cases were evaluated according to the WHO 2016 classification, using morphological criteria and an appropriate immunohistochemical panel. All of the samples had a follicular pattern of neoplastic lymphoid infiltrate and the follicular dendritic cell meshworks were present in follicles. Based on these findings transformation to DLBCL was excluded. All available clinical and pathological data of the FL patients were obtained from the online clinical database of DEU hospital. **Table 1** shows the clinicopathological characteristics (e.g. type of therapy, cancer stage, the percentage of Ki-67^+^ cells) of the FL cases. In FDG PET/CT imaging, malignant tissues can be distinguished with their higher uptake of FDG than normal. Briefly, FDG PET/CT was performed as follows: Six hours after starvation, F-18 FDG was applied intravenously to FL patients. With the purpose of attenuation correction, low dose CT and then PET images were obtained 60 mins post-injection. The number of anatomical regions potentially having tumor involvement was identified from the FDG PET/CT reports based on their SUVmax measurements.

**Table 1:** Clinicopathological characteristics of the FL cases.

	Asymptomatic (N = 7)	Symptomatic (N = 13)
**Gender**		
Female	6 (85.7%)	9 (69.2%)
Male	1 (14.3%)	4 (30.8%)
**Age (years)**		
Mean (SD)	54.4 (17.4)	50.2 (10.6)
Median [Min, Max]	58.0 [32.0, 78.0]	54.0 [34.0, 62.0]
**Stage**		
I	1 (14.3%)	1 (7.7%)
II	2 (28.6%)	3 (23.1%)
III	3 (42.9%)	3 (23.1%)
IV	1 (14.3%)	6 (46.2%)
**Ki67 Index (%)**		
Mean (SD)	23.0 (2.76)	35.5 (10.9)
Median [Min, Max]	23.0 [20.0, 27.0]	35.0 [20.0, 55.0]
Missing	1 (14.3%)	2 (15.4%)
**LDH Level (U/l)**		
Mean (SD)	201 (28.0)	238 (101)
Median [Min, Max]	205 [170, 251]	224 [110, 518]
**Treatment**		
Watch-and-Wait	7 (100%)	0 (0%)
R-Bendamustine	0 (0%)	3 (23.1%)
R-CHOP	0 (0%)	8 (61.5%)
R-CVP	0 (0%)	1 (7.7%)
R-CVP-Radiotherapy	0 (0%)	1 (7.7%)

LDH, Lactate dehydrogenase.

R-Bendamustine, Rituximab-Bendamustine.

R-CHOP, Rituximab- Cyclophosphamide, Hydroxydaunomycin, Oncovin, Prednisone.

R-CVP, Rituximab- Cyclophosphamide, Vincristine Sulfate, Prednisone.

### Plasma cfDNA and Granulocyte DNA Isolation From Peripheral Blood of FL Patients

Peripheral blood was collected from newly diagnosed 13 symptomatic and 7 asymptomatic FL cases before treatment. From 12 of 13 symptomatic FL cases, peripheral blood was collected second time after treatment as one FL patient passed away. The treatment and timeframe for peripheral blood samplings are shown in **Supplementary Table S1**. For each sample, 12 ml of peripheral blood was withdrawn into K2 EDTA tubes (BD Vacutainer, USA) in the department of hematology at DEU, and placed immediately on ice. Blood samples were transferred into 15 ml tubes, and centrifuged at 1600g for 10 min at 4°C. Supernatants were transferred to 1.5 ml tubes with each containing 1 ml plasma. The tubes were spun down to remove cellular debris at 16.000g for 5 min at 4°C. The supernatants were then transferred into new tubes, and stored in the -80°C freezer until cfDNA isolations. Circulating cell-free DNA (cfDNA) isolation was performed using QIAamp Circulating Nucleic Acid Kit (Qiagen Inc., Germany) according to the manufacturer’s recommendations. The size distribution of plasma cfDNA samples were evaluated with Agilent Bioanalyzer 2100 system to ensure fragment size distribution at around 150 bp (**Supplementary Figure S1**), which is the typical size for circulating cell-free DNA (22). Granulocytes was obtained from the pellet part of the blood sample by using Ficoll^®^ Paque Plus (MilliporeSigma, Burlington, MA) per the manufacturer’s instructions. After that, genomic DNA was isolated from granulocytes using PAXgene Blood DNA Kit (Qiagen Inc., Germany).

### Determination of cfDNA Concentrations in the Plasma of Treatment-Naive Follicular Lymphoma Patients

Concentrations of cfDNA isolated with QIAamp Circulating Nucleic Acid Kit were determined by using Qubit^®^ DNA Assay Kit and Qubit^®^ 2.0 Fluorometer. Plasma cfDNA concentrations per 1 ml of plasma were calculated using the following formula: (Qubit concentration measurement as ng/µl * elution volume as µl)/(plasma volume as ml used during cfDNA isolations).

### DNA Isolation From Formalin-Fixed Paraffin-Embedded Tumor Tissue Samples

Five µm-thick sections were prepared from tumor tissue blocks that were fixed with formalin and embedded in paraffin for each FL patient. The sections were then placed into 1.5 ml tubes, and stored at 4°C until isolations to prevent nucleic acid degradation (23). Tumor tissue genomic DNA samples were isolated using QIAamp DNA FFPE Tissue Kit (Qiagen Inc., Hilden, Germany) according to the manufacturer’s instructions.

### Custom-Designed Targeted Sequencing Gene Panel

The custom-designed NGS panel including FL and transformed FL-associated 110 genes with somatic mutations was constructed based on previously published studies (24–31). In this targeted NGS panel 575.481 bp genomic region, including the coding sequences and 50 bp flanking intronic sequences of 110 FL-related genes was targeted. FL-associated genes included in the targeted NGS panel are shown in **Table 2**.

**Table 2:** The list of 110 genes included in ultra-deep targeted sequencing of follicular lymphoma DNA samples.

ABCC10^b^	BCL6^b^	CCNK^b^	DTX1^b^	FOXO1^b^	HIST1H2BC^e^	KMT2D^b^	NEB^f^	SLC9A6^f^	UPF1^b^
AGTPBP1^b^	BCL7A^b^	CD40^f^	EBF1^e^	GNA13^b^	HIST1H2BD^e^	KNDC1^b^	NOTCH2^c^	SOCS1^e^	USP6^f^
ARHGEF1^b^	BRWD3^f^	CD79A^e^	EEF1A1^b^	GNAI2^b^	HIST1H2BG^e^	LAMP1^b^	NOTCH1^c^	SP140^b^	VPS39^d^
ARID1A^b^	BTG1^b^	CD79B^e^	ENPP3^f^	GNE^f^	IKZF2^f^	LPHN1^b^	POU2F2^d^	STAT3^e^	WDR64^d^
ATP6AP1^b^	C4orf49^f^	CEP112^f^	ENTPD4^f^	GRM7^d^	IKZF3^e^	LYST^d^	OR2M3^f^	STAT6^e^	ZCCHC6^b^
ATP6V1B2^b^	CALR^f^	CHRM3^d^	EP300^b^	HIST1H1B^e^	IL13RA1^b^	MATN2^f^	PCLO^d^	SVIL^b^	ZNF141^b^
AUTS2^f^	CARD11^e^	CPNE1^b^	ERBB2^f^	HIST1H1C^e^	IRF8^d^	MCL1^d^	PEX14^f^	TLR1^f^	ZNF236^b^
B2M^b^	CASC5^b^	CREBBP^e^	ETV1^b^	HIST1H1D^e^	KIR2DL3^f^	MEF2B^b^	PLCG2^e^	TNFAIP3^e^	ZNF423^b^
BAZ2B^f^	CASZ1^b^	CTSS^f^	EZH2^g^	HIST1H1E^e^	KLHDC7B^d^	MON2^b^	PRKCB^e^	TNFRSF14^e^	ZNF541^d^
BCL10^e^	CCAR1^f^	DIRAS3^f^	FAT2^f^	HIST1H2AC^e^	KLHL6^b^	MUC4^f^	RBM23^b^	TP53^h^	ZNF595^b^
BCL2^a^	CCND3^b^	DMRT3^d^	FN1^b^	HIST1H2AG^e^	KMT2C^b^	MYD88^e^	ROS1^f^	TRAFD1^b^	ZNF672^b^

^a^, (24); ^b^, (25); ^c^, (26); ^d^, (27); ^e^, (28); ^f^, (29); ^g^, (30); ^h^, (31).

The genes reported to be mutated in FL tumor tissues in 8 previous research articles are shown in alphabetical order.

### Library Preparations and Ultra-Deep Next-Generation Sequencing

The targeted sequencing experiments on plasma cfDNA, tumor tissue DNA, and granulocyte gDNA samples from 20 diagnostic FL cases, and 12 plasma cfDNA samples of immunochemotherapy treated FL cases were performed in Novogene Genome Sequencing Company (Hong Kong, China). These target regions were captured with 6385 probes; and Agilent SureselectXT Custom Kit was used for library preparations. Captured regions were paired-end sequenced using a HiSeq system, and 150 bp NGS reads were generated. Targeted genomic regions were covered with > 1500X in-depth for the identification of somatic variants with low variant allele fractions (VAF).

### Computational Bioinformatics Analyses for Somatic Variant Identification

Somatic variants were identified by using a computational bioinformatics pipeline that includes the MuTect2 variant caller (**Supplementary Figure S2**). The workflow of this pipeline is briefly as follows: First, the quality of raw NGS reads were evaluated with the FASTQC program (https://www.bioinformatics.babraham.ac.uk/projects/fastqc/). Secondly, the quality of raw NGS reads were improved by applying the AfterQC program (32). Processed NGS reads were then aligned to the human reference genome (hg19) with the BWA-MEM tool (33). Picard tool (https://broadinstitute.github.io/picard/) was used to mark and filter off the PCR duplicates in NGS reads processed with SAMtools (34). MuTect2 variant caller, which can identify somatic variants with a low percentage of VAF, was used to identify somatic variants in plasma cfDNA and tumor tissue DNA samples (35). Patient-matched granulocyte gDNA samples were used to filter off the germline SNPs. Identified variants were annotated to genomic regions with Annovar (36). In the final stage, the aligned NGS reads corresponding to the genomic locations of the identified variants were visually investigated with Integrative Genomics Viewer (IGV) to verify the presence and somatic nature of the mutations (37). During IGV visualizations, the following inclusion criteria were applied for the identification of a confident list of somatic variants: 1) Variants present in the COSMIC database (release v89), and associated with hematopoietic and lymphoid tissues; 2) Missense, nonsense, indel, or splice site mutations; 3) Variants with a VAF **>** 0.5%; 4) Variants in cfDNA or tumor tissue DNA with a VAF > 4 fold compared to that in the granulocyte gDNA; and 5) Variants with >5 forward and >5 reverse NGS reads.

### PCR-Sanger Sequencing

PCR-Sanger was applied on FL cfDNA and/or tumor tissue DNA to cross-validate selected somatic variants with a percentage of VAF greater than 20%. The DNA nucleotide sequences nearby the selected variants were obtained using the “Get DNA” bioinformatics tool available in UCSC Genome Browser (http://genome.ucsc.edu/). The primer pairs to be used for amplification of the genomic regions including the evaluated variants were designed with the PrimerQuest software (IDT DNA technologies, Coralville, IA). Designed primers were produced by Sentebiolab Biotech (Ankara, Turkey). Optimum annealing temperatures of the primer pairs were determined by gradient PCR. FL cfDNA and tumor tissue DNA samples were amplified with PCR and then send to Eurofins Genomics (Ebersberg, Germany), where PCR purifications and Sanger sequencing reactions were performed with forward and reverse primers. Vector NTI 10.3.0 (Invitrogen, Carlsbad, CA) was used to detect mutations in sequenced FL DNA samples by comparing to the human reference gene sequences. The primers used in PCR-Sanger experiments are shown in **Supplementary Table S2**.

### Statistical Analysis

RStudio software (https://www.rstudio.com/) was used for linear regression, survival, and signaling pathway analyses. Survival (38) and Survminer (39) R packages were used during survival analyses and generation of Kaplan-Meier graphics. The statistical significance of the differences in survival of FL patients were evaluated with the log-rank test. Cutoff level for high and low values for each evaluated quantitative variable (i.e. cfDNA concentrations, serum LDH levels, Ki-67 index, and FDG PET/CT positive regions) were determined with maximally selected rank statistics using the “surv_cutpoint” function of the Survminer R package. The cutoff levels classifying low and high cfDNA groups were 17 ng/µl for overall survival and 16 ng/µl for progression-free survival analyses. These cutoff levels were 35% for Ki-67 index, 262.0 units per liter for LDH level, and 10 regions for FDG PET/CT positive regions. The R-value of linear regression analyses for the percentage of VAFs was calculated on the Spearman correlation model; and the p-value was generated with t-test. Hypergeometric distribution test was used to determine the statistical significance of enriched pathways by using the ReactomePA package (40). p<0.05 was considered statistically significant for the analyses performed with the RStudio software.

## Results

### Overall Plan of the Study

The overall plan of the study involves the following steps: 1) Construction of a custom-designed targeted NGS panel based on previously mutated genes in FL and tFL; 2) Collection of peripheral blood and tumor tissue samples from treatment-naive and immunochemotherapy-treated FL patients; 3) DNA isolations from peripheral blood plasma, granulocyte, and FFPE tumor tissue samples; 4) Identification of cancer-associated mutations through computational bioinformatics analyses; 5) Investigation of the mutual presence of somatic variants in plasma cfDNA and tumor tissue DNA; 6) Survival analyses with identified variants; 7) PCR-Sanger cross-validation of selected variants. The workflow of this study is shown in **Supplementary Figure S3**.

### Basic Statistics for Ultra-Deep Targeted Next-Generation Sequencing

Full set of plasma cfDNA, tumor tissue gDNA, granulocyte gDNA from 20 treatment-naive FL cases (n=60) as well as 12 post-treatment plasma cfDNA samples passed the quality parameters. The average basic NGS parameters per FL DNA samples are as follows: Total raw reads: 31.7*10^^6^ ± 12.3*10^^6^, raw data: 9.5 ± 3.7 gigabyte, the percentage of clean reads to raw reads: 93.9% ± 7.8, the average error rate in all bases: 0.011% ± 0.003, Q20: 96.8% ± 0.6, Q30: 92.5% ± 1.3, and GC content: 48.5% ± 2.2. Basic ultra-deep targeted sequencing statistics are shown in **Supplementary Table S3**.

### The Impact of cfDNA Concentrations on Survival of Follicular Lymphoma Patients

**Figure 1:**
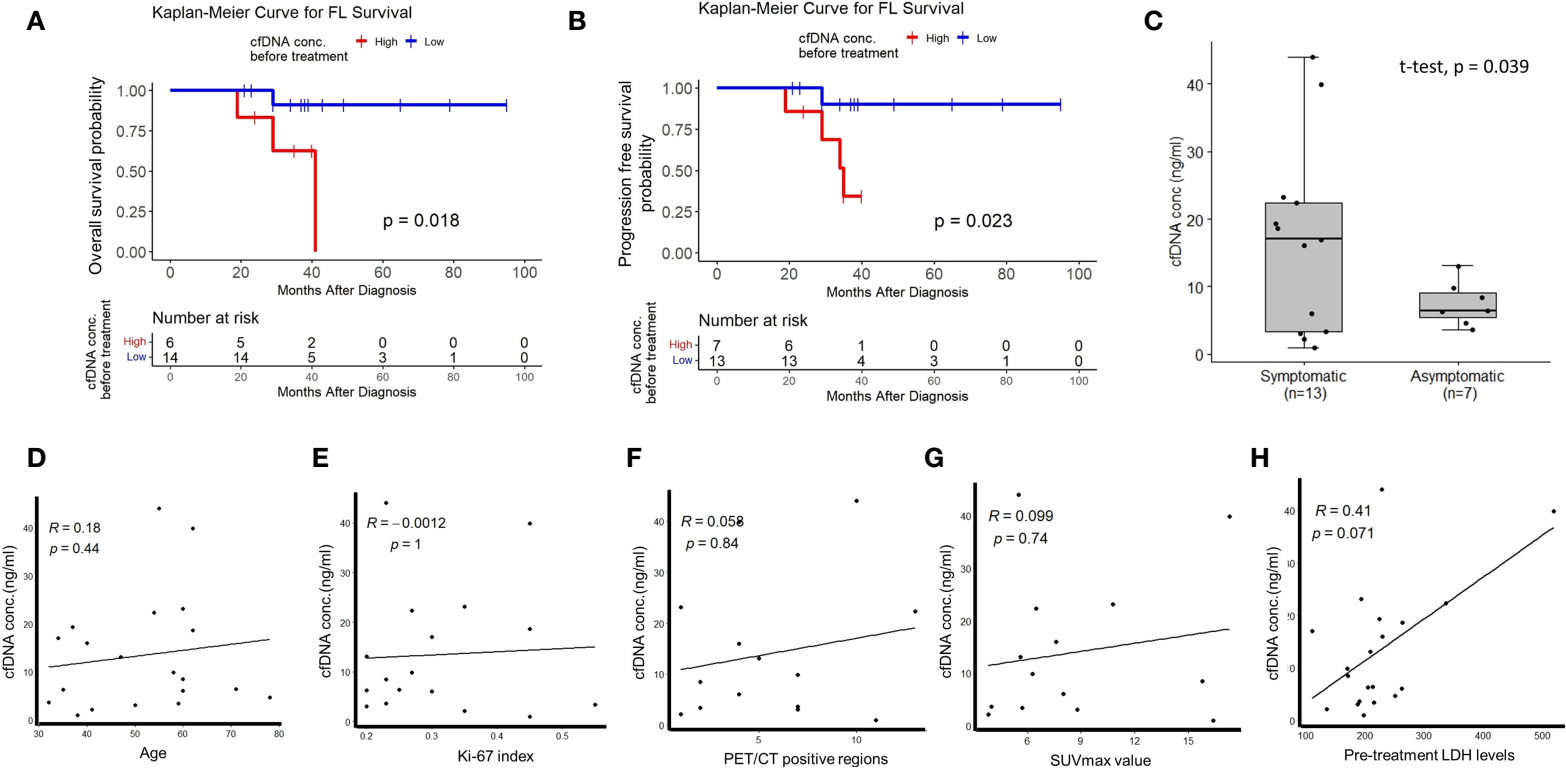
The relationship between high plasma cfDNA concentrations and clinical variables in treatment-naive FL patients. Kaplan-Meier plots showing the relationship between high plasma cfDNA concentrations and overall survival **(A)** or progression-free survival **(B)** in treatment-naive follicular lymphoma patients. **(C)** Box-whisker plot comparing plasma cfDNA concentrations in symptomatic and asymptomatic FL patients. Linear regression graphics showing the relationship between plasma cfDNA concentrations and age **(D)**, Ki-67 index **(E)**, PET-CT positive region number **(F)**, SUVmax value **(G)**, and LDH levels **(H)**.

To address whether plasma cfDNA concentrations have any relationship with FL patient prognosis or not, we compared survival of treatment-naive FL patients stratified based on plasma cfDNA concentrations. We observed significantly inferior overall survival (**Figure 1A**) and progression-free survival (**Figure 1B**) of FL patients with high plasma cfDNA concentrations compared to those having low cfDNA concentrations. Importantly, plasma cfDNA concentrations of symptomatic FL patients were significantly higher compared to those of asymptomatic ones (**Figure 1C**). Next, by performing linear regression analyses we evaluated whether plasma cfDNA concentrations correlate with demographic, clinical, or pathological parameters (i.e. age, Ki-67 index, pre-treatment LDH levels, FDG PET/CT positive regions, or SUVmax value) or not. We did not observe any correlation between plasma cfDNA levels and age, Ki-67 index, FDG PET/CT positive regions, SUVmax value, or LDH levels (**Figures 1D–H**).

### The Numbers and the Extend of Overlap of Mutations in cfDNA and Tumor Tissue DNA

**Figure 2:**
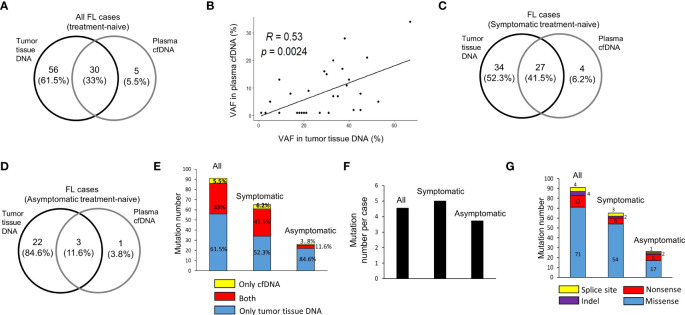
Cancer-associated somatic variant numbers in DNA samples of FL patients. **(A)** The numbers of somatic mutations detected in plasma cfDNA and tumor tissue DNA for all FL patients shown as intersecting Venn diagrams. **(B)** Spearman correlation analysis between variant allele fractions (VAF) of mutations detected in both plasma cfDNA and tumor tissue DNA samples. The numbers and percentages of somatic mutations detected in plasma cfDNA and tumor tissue DNA for symptomatic **(C)** and asymptomatic **(D)** FL patients shown as intersecting Venn diagrams. **(E)** Bar graph of cancer-associated somatic mutation number per patient for all, symptomatic, or asymptomatic FL patients. **(F)** Bar graph of total numbers of somatic mutations detected in cfDNA and/or tumor tissue DNA for symptomatic and asymptomatic FL patients. **(G)** Bar graph of total number of somatic missense, nonsense, indel and splice site mutations detected in 13 symptomatic and 7 asymptomatic FL cases.

In 20 treatment-naive FL patients, in total 91 hematological cancer-associated somatic variants were detected in cfDNA and/or tumor tissue DNA samples for 31 of 110 FL-related genes targeted. Among these variants, 30 (33%) of them were common in both plasma cfDNA and tumor tissue DNA, whereas 56 (61.5%) and 5 (5.5%) of them were observed only in tumor tissue DNA or plasma cfDNA, respectively (**Figure 2A**). Of significance, we observed a significant positive correlation (R=0.53, p=0.002) between variant allele fractions of variants in tumor tissues and plasma cfDNA samples (**Figure 2B**). Next, we comparatively analyzed the number and percentage of mutations in cfDNA and/or tumor tissue DNA of symptomatic and asymptomatic FL cases. Among 91 cancer-associated somatic variants, 65 of them were identified in symptomatic, and 26 of them were identified in asymptomatic cases. Twenty seven (41.5%) of these 65 mutations were observed in both cfDNA and tumor tissue DNA, 34 (52.3%) of them were observed only in tumor tissue DNA, and four (6.2%) of them were present only in cfDNA (**Figure 2C**). In asymptomatic FL patients, 3 (11.6%) of 26 somatic mutations were observed in both cfDNA and tumor tissue DNA, 22 (84.6%) of them only in tumor tissue DNA, and just one (3.8%) of them were present only in cfDNA (**Figure 2D**). The proportion of somatic mutations present in cfDNA samples was markedly higher in symptomatic cases and most of the variants detected in asymptomatic cases were only present in tumor tissue DNA (**Figure 2E**). Importantly, we observed at least one variant present in both DNA samples in 10 of 13 (77%) of symptomatic FL cases (**Supplementary Figure S4**) and in 1 of 7 (14.3%) of asymptomatic cases (**Supplementary Figure S5**). When we compared the number of mutations per FL case in these two FL groups, we observed that the number of mutations per FL patient was marginally higher in symptomatic cases (average: 5.0; median: 4.0) compared to asymptomatic cases (average: 3.7; median: 3.0) (**Figure 2F**). In 65 mutations observed in symptomatic cases there were 54 missense, 6 nonsense, 2 indel, and 3 splice site mutations. In 26 mutations observed in asymptomatic cases there were 17 missense, 6 nonsense, 2 indel, and only one splice site mutations (**Figure 2G**). The details of 91 cancer-associated somatic mutations identified through ultra-deep targeted NGS are shown in **Supplementary Table S4**.

### Mutated Genes and the Mutation Types Observed in cfDNA and Tumor Tissue DNA of Follicular Lymphoma Cases

**Figure 3:**
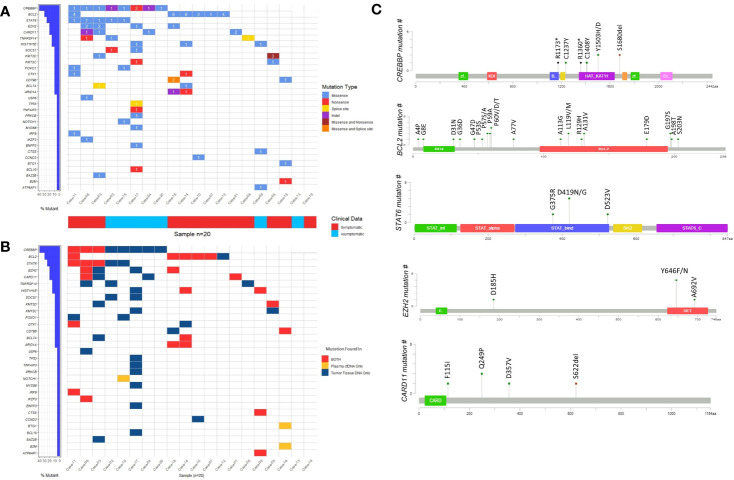
The somatic variants identified by ultra-deep sequencing in cfDNA and/or tumor tissue DNA FL patients. **(A)** Waterfall plot showing the genes with missense, nonsense, splice site, and indel mutations detected in FL cases. Number labels indicate the number of mutations found on that gene for that FL case. Horizontal blue bars on the left show the percentage of FL cases mutated for the genes. If a gene displayed as having two mutation types includes three or more mutations in a FL case, it means at least one of these mutations belong to a mutation type different than others. **(B)** Waterfall plot showing the DNA type mutations found in plasma cfDNA, FFPE tDNA or both. Horizontal blue bars on the left show the percentage of FL cases mutated for the genes. If a gene in a FL case includes more than one mutation that is displayed as present in both DNA types, it means at least one of these mutations is present in both plasma cfDNA and FFPE tumor tissue DNA samples. **(C)** The domains and affected residues of recurrently mutated genes in FL cases determined with Cbioportal mutation mapper.

To address whether genes reported to be recurrently mutated in previous studies are also frequently mutated in our cohort of FL patients, we visually observed the frequency of the mutated genes as well as the presence of these genes in different DNA types by generating waterfall graphics (**Figures 3A, B** **)**. The genes mutated in at least 20% of 20 FL cases are as follows: *CREBBP* (40%), *BCL2* (30%), *STAT6* (25%), *CARD11* (20%), and *EZH2* (20% of cases) **(** **Figure 3A** **)**. Of note, more than one mutation per gene was observed in some FL cases. *CREBBP* mutations were generally located on the HAT/KAT domain (**Figure 3C**). Mutations of *BCL2* were distributed in different parts of the protein whereas *STAT6* mutations were enriched in the STAT binding domain, and the *EZH2* mutations were mainly on the SET domain (**Figure 3C**). Among five cancer-associated STAT6 missense variants, three of them (i.e. p.D419G, p.D419N, and p.D523V) were present in both cfDNA and tumor tissue DNA in FL patients. Moreover, all patients with *STAT6* mutations were also carrying *CREBBP* mutations consistent with the observations in a previously reported study (41). In total 6 *EZH2* variants were identified in four FL patients, of which two were present in both cfDNA and tumor tissue DNA. In addition, we identified the enriched signaling pathways and biological processes associated with the 31 genes identified to have somatic variations in cfDNA and/or tumor tissue DNA samples. This analysis showed that mutated genes are mainly enriched in “Transcriptional regulation by RUNX1”, “IL-4 and IL-13 signaling”, “Chromatin modifying genes”, and “Signaling by the B-cell receptor” (**Supplementary Figure S6**). However, it should be noted that the number of genes used as input for the gene ontology analysis is low, which may result in overestimation of the signaling pathways or biological processes identified.

We then asked whether any of the somatic variations identified in diagnostic FL cases persisted after immunochemotherapy treatment in symptomatic FL patients. Sixty-five somatic mutations were identified in cfDNA and/or FFPE tumor tissue DNA in 13 symptomatic FL cases. We could not obtain plasma cfDNA from one of the FL cases (i.e. Case-10) as this patient passed away. Based on targeted ultra-deep NGS analyses, 9 of these 12 symptomatic FL cases had at least one somatic mutation in their plasma cfDNA before treatment started. The total number of somatic mutations detectable in pre-treatment plasma cfDNA of these 9 symptomatic FL cases was 30. We specifically evaluated these 30 mutations in cfDNA samples after immunochemotherapy. In plasma cfDNA samples obtained post-therapy, only one indel (i.e. CARD11 p.S622delS, %VAF: 0.8) among 30 missense, nonsense, and indel variant was detectable based on our stringent variant identification criteria. Of note, there was no significant difference in plasma cfDNA concentrations between treatment-naive and post-therapy FL samples (**Supplementary Figure S7**).

### The Influence of *BCL2* Mutations on Survival of Follicular Lymphoma Patients

**Figure 4:**
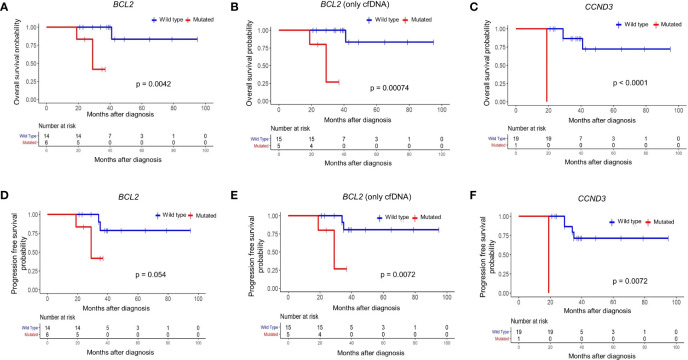
*BCL2* mutations in plasma cfDNA predict poor prognosis in follicular lymphoma. Kaplan-Meier plots showing overall survival for FL patients based on mutation status of *BCL2* gene in cfDNA and/or tumor tissue DNA **(A)**, *BCL2* gene in only cfDNA **(B)**, or *CCND3* gene in cfDNA and/or tumor tissue DNA **(C)**. Kaplan-Meier plots showing progression-free survival for FL patients based on mutation status of *BCL2* gene in cfDNA and/or tumor tissue DNA **(D)**, *BCL2* gene in only cfDNA **(E)**, or *CCND3* gene in cfDNA and/or tumor tissue DNA **(F)**. p-values represent the statistical significance based on the log-rank test.

To address whether the identified mutations predict overall and progression-free survival of FL patients, we applied survival analyses for each of 31 mutated genes with or without mutations in cfDNA and/or tumor tissue DNA samples. When we evaluated mutations detected in cfDNA and/or tumor tissue DNA, mutations in the *BCL2* gene predicted a poor overall survival (p = 0.0042) (**Figure 4A**). Similarly, when FL patients are dichotomized based on the presence or absence of *BCL2* mutations only in plasma cfDNA, FL patients with *BCL2* mutations were significantly (p = 0.0007) associated with inferior overall survival (**Figure 4B**). We also observed poor overall survival (p < 0.0001) of a CCND3-mutated FL patient (**Figure 4C**); however, CCND3 was mutated only in the tumor tissue of this FL case. When FL cases were dichotomized based on the presence of *BCL2* mutations in cfDNA and/or tumor tissue DNA, *BCL2*-mutated FL cases were associated with poor progression-free survival albeit not statistically significant (p = 0.05) (**Figure 4D**). Importantly, FL patients with *BCL2* mutations were significantly associated with progression-free survival when only cfDNA mutation status was taken into account (**Figure 4E**). Finally, a *CCND3*-mutated FL case showed significantly poor progression-free survival (**Figure 4F**). When Fisher’s exact test was used to evaluate the statistical significance of these survival differences, presence of *BCL2* mutation in cfDNA was still associated with poor FL survival; whereas, no statistical significance was observed in terms of survival for the CCND3-mutated FL case (**Supplementary Table S5**). No association with FL patient survival was detected for other mutated genes evaluated. Kaplan-Meier curves for the recurrently (15% or more of FL cases) mutated genes with no association to progression-free survival are shown in **Supplementary Figure S8**.

### The Effect of Co-Presence of *BCL2* Mutations and Adverse Clinical Variables on Follicular Lymphoma Survival

Next we evaluated the relationship between previously established clinicopathological variables (i.e. Ki-67 index, serum LDH levels, PET/CT positivity) routinely used in prognostic evaluations during FL diagnosis on patient survival. As expected, high expression of Ki-67 (p = 0.013), high serum LDH levels (p = 0.00076), and high number of FDG PET/CT positive regions (p = 0.00014) were significantly associated with inferior prognosis (**Supplementary Figure S9**). To address whether *BCL2* genotyping can improve current prognostic evaluations in a non-invasive fashion, we evaluated the co-presence of *BCL2* mutations in plasma cfDNA and adverse clinical variables on predicting overall and progression-free survival. Of significance, FL patients carrying *BCL2* mutation with high Ki-67 index (**Supplementary Figure S10A**), high LDH level (**Supplementary Figure S10B**), or high number of positive FDG PET/CT regions (**Supplementary Figure S10C**) showed the worst overall survival. Similarly, FL patients with *BCL2* mutations and high value for one of these parameters had the most inferior progression-free survival (**Supplementary Figures S10D–F**). The relationship between FL patient survival and different variables (*BCL2* mutation status, clinical variables, different treatment types) are altogether shown in **Table 3**.

**Table 3:** The relationship between clinical variables as well as *BCL2* or *CCND3* mutation status and follicular lymphoma survival.

Variable		Number	Event	OS (%)	*p*	Event	PFS (%)	*p*
Ki-67 percentage (%)	> 35%	4	3	25.0	0.014	3	25.0	0.013
	≤ 35%	13	1	92.3		2	84.6	
LDH level before therapy (units/L)	> 262 units/L	3	3	0.0	0.00049	3	0.0	0.00076
	≤ 262 units/L	17	1	94.1		2	88.2	
Number of positive FDG PET/CT regions	> 10	2	2	0.0	0.00014	2	0.0	0.00014
	≤ 10	12	1	91.7		1	91.7	
cfDNA concentration before therapy (ng/ml)	> 16 ng/ul	6	3	50.0	0.018	4	33.3	0.023
	≤ 16 ng/ul	14	1	92.9		1	92.9	
*BCL2* gene	Mutated	6	3	50.0	0.0042	3	50.0	0.054
	Wild Type	14	1	92.9		2	85.7	
*CCND3* gene	Mutated	1	1	0.0	<0.0001	1	0.0	<0.0001
	Wild Type	19	3	84.2		4	78.9	
*BCL2* gene and Ki-67 percentage (%)	Mutated and High (> 35)	2	2	0	0.0037	2	0	0.0082
	Mutated and Low (≤ 35) or Wild Type and High (> 35)	5	2	60		2	60	
	Wild Type and Low (≤ 35)	10	0	100		1	90	
*BCL2* gene and LDH level before therapy (units/L)	Mutated and High (> 262)	2	2	0	<0.0001	2	0	0.00013
	Mutated and Low (≤ 262) or Wild Type and High (> 262)	5	2	60		2	60	
	Wild Type and Low (≤ 262)	13	0	100		1	92.3	
*BCL2* gene and Number of positive FDG PET/CT regions	Mutated and High (> 10)	2	2	0	0.0007	2	0	0.00067
	Mutated and Low (≤ 10) or Wild Type and High (> 10)	2	0	100		0	100	
	Wild Type and Low (≤ 10)	10	1	90		1	90	
Treatment	R-Bendamustine	3	1	66.7	0.42	1	66.7	0.47
	R-CHOP	8	3	62.5		4	50.0	
	R-CVP	2	0	100.0		0	100.0	

OS, Overall Survival; PFS, Progression-Free Survival.

### Histological Transformation-Associated *BTG1* and *B2M* Mutations Were Exclusively Present in the cfDNA of a FL Patient

**Figure 5:**
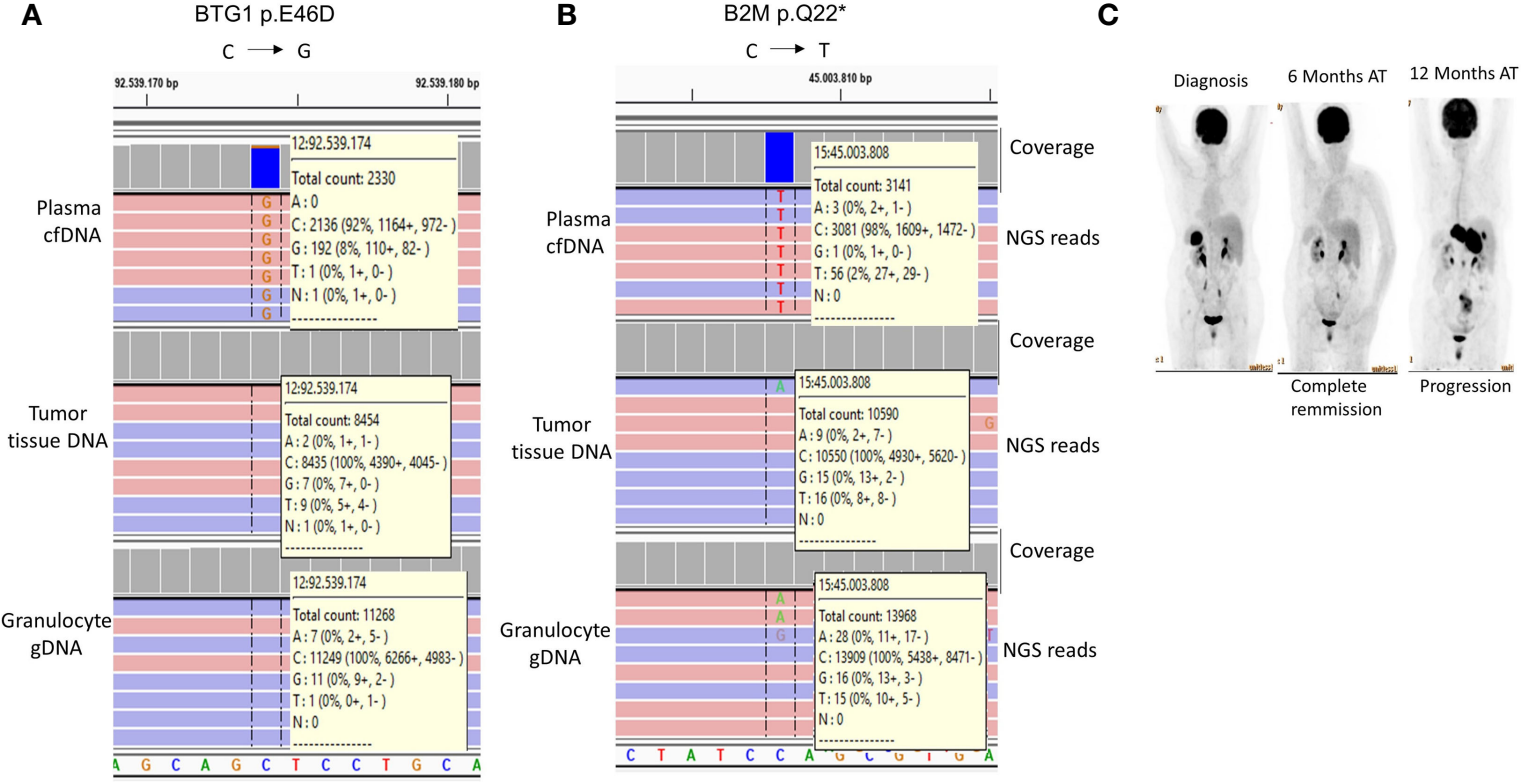
Histological transformation-associated *BTG1* and *B2M* mutations detected exclusively in cfDNA of a FL patient. IGV snapshots showing BTG1 p.E46D **(A)** and B2M p.Q22* **(B)** mutations in the plasma cfDNA sample of a FL patient (Case-16). The mutation status of the patient-matched granulocyte gDNA is also shown as a negative control sample. **(C)** FDG PET/CT images of Case-16 are shown before and after immunochemotherapy. AT, After therapy.

It has been recently shown that mutations in certain genes including those of *BTG1* and *B2M* could identify FL cases with a tendency for early progression and histological transformation (42). In our study, one FL case (Case-16) had the BTG1 p.E46D (**Figure 5A**) and B2M p.Q22* (**Figure 5B**) mutations specifically in plasma cfDNA but not in the tumor tissue DNA of the same FL patient. As expected, patient-matched granulocyte gDNA did not have variant reads, which shows the somatic origin of these mutations. PET/CT imaging showed complete remission 6 months after R-CHOP immunochemotherapy; however, Case-16 showed progressive disease 12 months post-therapy (**Figure 5C**). The observations with this FL case suggest the possibility of identification of high-risk FL patients non-invasively during diagnosis through cfDNA genotyping.

### Cross-Validation of Selected Variants With PCR-Sanger Sequencing

Next, we performed cross-validation of selected variants detected with ultra-deep targeted sequencing using PCR-Sanger. As the threshold for the percentage of VAF for Sanger sequencing is 20%, we chose the variants with a VAF percentage higher than 20%. Cross-validation of selected somatic variants using PCR-Sanger showed 100% consistency with NGS results (**Table 4**). PCR-Sanger cross-validation of ARID1A p.R1722* variant identified in plasma cfDNA and tumor tissue DNA is shown in **Supplementary Figure S11** as a representative.

**Table 4:** List of genes and variants cross-validated with PCR-Sanger sequencing and their confirmation status.

Case number	Gene	Sample type	Nucleotide change	AA change	Mutation detection	NGS VAF percentage (%)
Case-11	CREBBP	FFPE tDNA	C > T	C1237Y	+	27
Case-14	HIST1H1E	Granulocyte	C > T	P131S	–	0
Case-14	HIST1H1E	FFPE tDNA	C > T	P131S	+	67
Case-14	HIST1H1E	cfDNA	C > T	P131S	+	34
Case-14	ARID1A	Granulocyte	C > T	R1722*	–	0
Case-14	ARID1A	FFPE tDNA	C > T	R1722*	+	33
Case-14	ARID1A	cfDNA	C > T	R1722*	+	20
Case-11	STAT6	Granulocyte	T > A	D523V	–	0
Case-11	STAT6	FFPE tDNA	T > A	D523V	+	45
Case-09	CTSS	Granulocyte	A > C	Y132D	–	0
Case-09	CTSS	FFPE tDNA	A > C	Y132D	+	39
Case-09	HIST1H1E	Granulocyte	G > C	A65P	–	0
Case-09	HIST1H1E	FFPE tDNA	G > C	A65P	+	53
Case-09	ATP6AP1	Granulocyte	G > A	G363R	–	0
Case-09	ATP6AP1	FFPE tDNA	G > A	G363R	+	45
Case-11	IRF8	Granulocyte	T > C	Y23H	–	0
Case-11	IRF8	FFPE tDNA	T > C	Y23H	+	31
Case-05	KMT2D	Granulocyte	G > A	R2417*	–	0
Case-05	KMT2D	FFPE tDNA	G > A	R2417*	+	33

AA, Amino acid.

NGS, Next-generation sequencing.

VAF, Variant allele fraction.

FFPE tDNA, Formalin-fixed paraffin-embedded tumor tissue DNA.

Granulocyte, Granulocyte gDNA.

## Discussion

Recurrent mutations of genes involved in epigenetic regulation (e.g. *KMT2D*, *CREBBP*, and *EZH2*), JAK-STAT pathway (e.g. *SOCS1*, *STAT6*), immune signaling (i.e. *TNFRSF14*), and BCR/NFKB signaling (e.g. *CARD11*, *CD79B*) were previously reported in follicular lymphoma cases (28). Consistent with these results, we observed recurrent mutations in *CREBBP*, *BCL2*, *STAT6*, *EZH2*, *CARD11*, *TNFRSF14*, *SOCS1*, and *KMT2D* in our FL cases with targeted next-generation sequencing (**Figure 3A**). Importantly, KMT2D variations reported to be associated with FL pathogenesis such as p.R2417* were observed in plasma cfDNA and tumor tissue DNA of our FL cases (**Supplementary Figure S12**) (43).

Previous studies revealed activating mutations of *STAT6* in the DNA binding domain, which is associated with FL pathogenesis (44). Given that in three FL patients activating STAT6 (i.e. p.D419G, p.D419N, and p.D523V) mutations present in tumor tissue DNA were detectable in plasma cfDNA, it is possible to apply therapies targeting the JAK-STAT6 pathway for the FL patients with these mutations in cfDNA. Of note, specific inhibitors of STAT6 or its signaling pathway are under development (45).

Importantly, four of the EZH2 variants were located on Y646, which is a known mutational hotspot (**Figure 3C**). H3K27 methyltransferase EZH2 gain-of-function mutations including Y646 were reported to be frequently observed as an early alteration in FL pathogenesis (9). A report showed anti-tumor efficacy for EPZ-6438, a selective inhibitor of EZH2, in Y646F-mutant xenograft tumors of non-Hodgkin lymphoma (46). Given that tazemetostat as an EZH2 inhibitor has shown activity against mutant EZH2 patients in a recent clinical trial (47), plasma cfDNA genotyping of pre-treatment FL patients may be used to decide on EZH2 targeting therapy.

Consistent with the previous studies revealing the prognostic utility of certain clinicopathological variables such as FDG PET/CT positivity (48) and serum LDH positivity (49), we observed poor survival of FL patients with high values for these variables. We also observed poor survival of FL patients with a high Ki-67 proliferation index despite the controversy on its prognostic value (50). Interestingly, in a previous study the prevalence of *BCL2* mutations detected in FL tumor tissue samples was 12% at diagnosis and 53% at the transformation; and FL patients with *BCL2* mutations had shortened survival and increased risk of histological transformation to aggressive B cell lymphomas (24). Observation of the prognosis-associated *BCL2* mutations for the first time in plasma cfDNA samples of FL cases have important implications for risk assessment as well as the guidance of therapy. Indeed, it may be more effective to choose aggressive therapies for the subset of FL patients having both *BCL2* mutations in cfDNA with any of these other poor prognostic indicators. However, validation in independent, larger cohorts of FL cases is needed to address these observations in our study with a relatively low number of cases. Furthermore, cross-validation of targeted NGS results of *BCL2* mutations having low VAFs in FL cfDNA with approaches more sensitive than Sanger sequencing (e.g. ddPCR (18)) will be one of the priorities of future research. The association of highly poor survival and the presence of *CCND3* mutations requires further investigation with larger FL patient cohorts as well to address whether prognostic *CCND3* mutations are observed in plasma cfDNA or not as we observed *CCND3* mutation in tumor tissue of only one FL case. Importantly, all seven genes of the m7-FLIPI (51) was included in our targeted NGS panel (**Table 2**). Given that none of the 5 mutated genes of m7-FLIPI (i.e. *ARID1A*, *CREBBP*, *CARD11*, *EZH2*, and *FOXO1*) showed association with survival in our FL patient cohort is consistent with a recent report indicating that its prognostic value in FL patients may be context dependent (52).

The marked difference observed between symptomatic and asymptomatic FL cases with respect to the percentage of mutations in cfDNA suggests that cfDNA genotyping may be more feasible for FL patients showing disease-associated symptoms. It should also be noted that 61.5% of the identified somatic mutations were specific to tumor tissues. This observation may be related to the relatively indolent character of follicular lymphomas compared to more aggressive lymphomas such as DLBCL where a higher proportion of tumor tissue-derived mutations was observed in patient-matched plasma cfDNA (21). However, we do not exclude the possibility that certain cancer-associated mutations in cfDNA of asymptomatic FL patients may have been missed due to the limitations of the targeted ultra-deep NGS approach applied in this study or the limited amount of cfDNA available from asymptomatic patients.

Identification of mutations in *BTG1* and *B2M* genes associated with early disease progression and/or histological transformation (42) exclusively in the plasma cfDNA sample of a FL case suggests that plasma cfDNA genotyping may provide additional prognostic information that may not be obtained from tumor tissues under certain circumstances. Of note, BTG1 p.E46D and B2M p.Q22* mutations were previously reported in two DLBCL cases, one of which was a transformed FL (43, 53). Our observation that the FL case with these mutations in cfDNA had a progressive disease is consistent with these previous reports. The lack of *BTG1* and *B2M* mutations in tumor tissue DNA suggests that ctDNA fragments with these mutations may have originated from distant metastatic tumor tissues with different genotypes in this FL patient. Nevertheless, it should also be noted that these mutations were observed in only one FL case; and plasma cfDNA genotyping of independent patient cohorts is needed to address whether these observations can be generalized for follicular lymphoma.

Given that many FL pathogenesis-associated mutations present in tumor tissues are also observed in patient-matched plasma cfDNA samples, it is possible -in principle- to detect these somatic mutations non-invasively with targeted next-generation sequencing during clinical applications. The positive correlation between the variant-allele fractions of mutations in patient-matched tumor tissue and plasma cfDNA implies that mutations in cfDNA originate from tumor tissues. As somatic mutations with VAF percentages >20% were cross-validated with Sanger sequencing in this study (**Supplementary Figure S11**), more sensitive methodologies such as ddPCR (18) or BEAMing (54) will be needed to specifically cross-validate these NGS-identified mutations with low percentages of VAFs in FL cases in the future.

In conclusion, we showed in this study the possibility that concentration measurement and/or genotyping of FL-related genes in plasma cfDNA of treatment-naive FL patients may be used -in principle- to improve current diagnostic and prognostic evaluations, thereby leading to more appropriate selection and timing for therapeutic interventions including targeted therapies.

## Data Availability Statement

All targeted NGS data files were submitted to the NCBI SRA database (accession no: PRJNA832096), and can be directly accessed through the following link: https://www.ncbi.nlm.nih.gov/sra/PRJNA832096.

## Ethics Statement

The studies involving human participants were reviewed and approved by Institutional Review Board of the Medical School at Dokuz Eylül University (protocol no: 2233-GOA). The patients/participants provided their written informed consent to participate in this study.

## Author Contributions

TH, HLY, XH, and BA: Performed research and analyzed data; EES and WZ: Computationally analyzed the targeted sequencing data; TÖS: Contributed to supervision of the computational bioinformatics analyses; AE and SÖ: Pathologically diagnosed the FL cases and provided tumor sections; AŞ, İA, AO, and MÖ: Clinically followed FL patients, provided the peripheral blood samples, compiled, and organized clinical data of the FL patients; TH and CK: Wrote the manuscript; HY. and CK: Financially supported the project; CK: Conceived the study, and supervised the project. All authors contributed to the article and approved the submitted version.

## Funding

This study was supported by TÜBİTAK (The Scientific and Technological Research Council of Turkey) 1001 program (no: 215S750) (CK), and the International Young Scientist Award from the National Natural Science Foundation of China (no: 81850410547) (CK, HY).

## Conflict of Interest

The authors declare that the research was conducted in the absence of any commercial or financial relationships that could be construed as a potential conflict of interest.

## Publisher’s Note

All claims expressed in this article are solely those of the authors and do not necessarily represent those of their affiliated organizations, or those of the publisher, the editors and the reviewers. Any product that may be evaluated in this article, or claim that may be made by its manufacturer, is not guaranteed or endorsed by the publisher.

## Abbreviations

cfDNA, Circulating cell-free DNA; ctDNA, Cell-free tumor DNA; DLBCL, Diffuse large B cell lymphoma; FDG PET/CT, 18F-fluorodeoxyglucose positron emission tomography/computed tomography; FFPE, Formalin-fixed paraffin-embedded; FL, Follicular lymphoma; gDNA, Genomic DNA; IGV, Integrative Genomics Viewer; LDH, Lactate dehydrogenase; NGS, Next-generation sequencing; R-CHOP, Rituximab-cyclophosphamide hydroxydaunorubicin-oncovin-prednisone; SUVmax, Maximum standard uptake value; VAF, Variant allele fraction.
