# Supplementary material for: Plasma Concentrations and Cancer-Associated Mutations in Cell-Free Circulating DNA of Treatment-Naive Follicular Lymphoma for Improved Non-Invasive Diagnosis and Prognosis
Source: Front Oncol. 2022 Jun 16;12:870487. doi: 10.3389/fonc.2022.870487 (PMC9252432; doi:10.3389/fonc.2022.870487)
Supplement: Supplementary file 1 [file DataSheet_1.docx]

**Supplementary Figures**

**
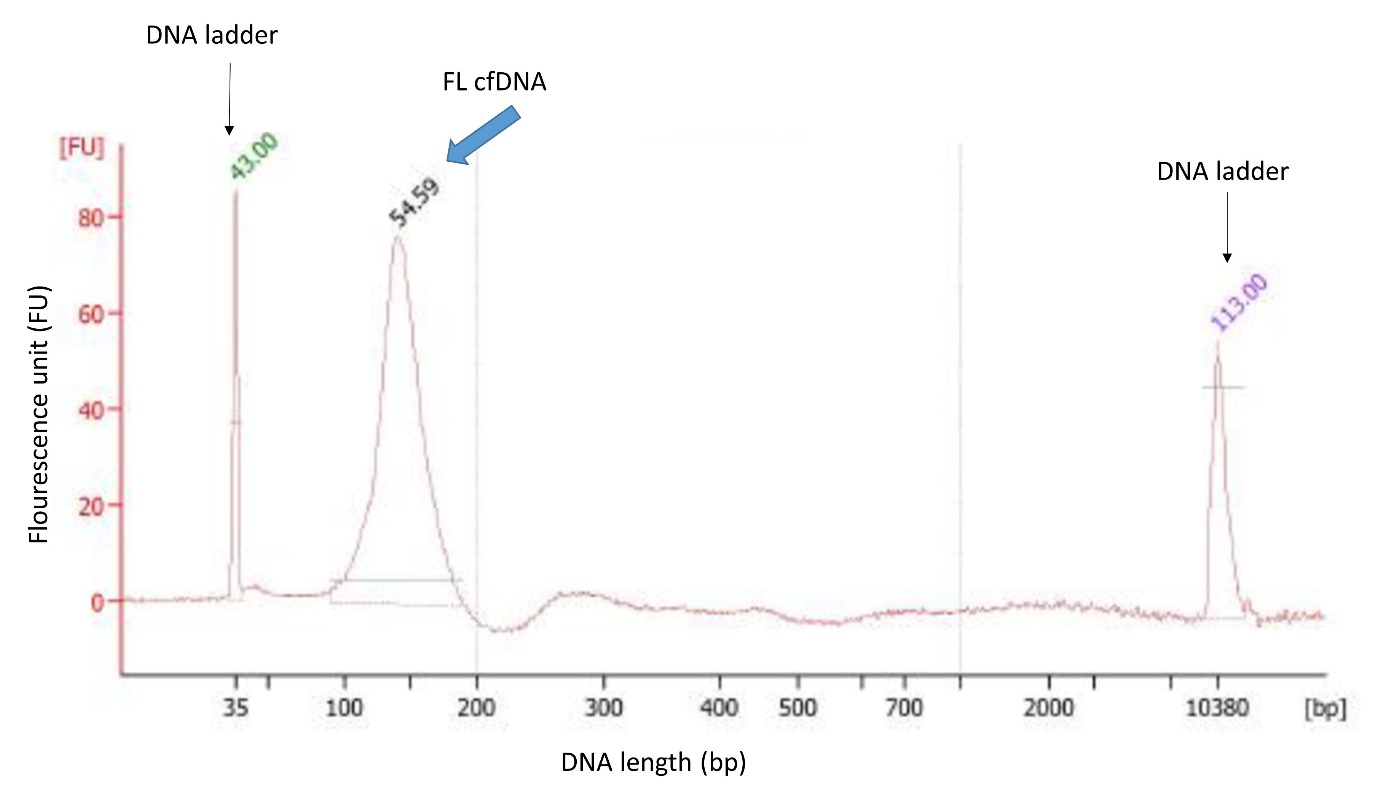
**

**Supplementary Figure S1. Plasma cfDNA length of a representative FL case.** Plasma cfDNA fragment sizes were evaluated with the Agilent Bioanalyzer 2100 system. DNA markers and the peak site for cfDNA fragments are shown with thin or blue color filled arrows, respectively. The X axis shows DNA fragment sizes and the Y axis shows fluorescence amount.


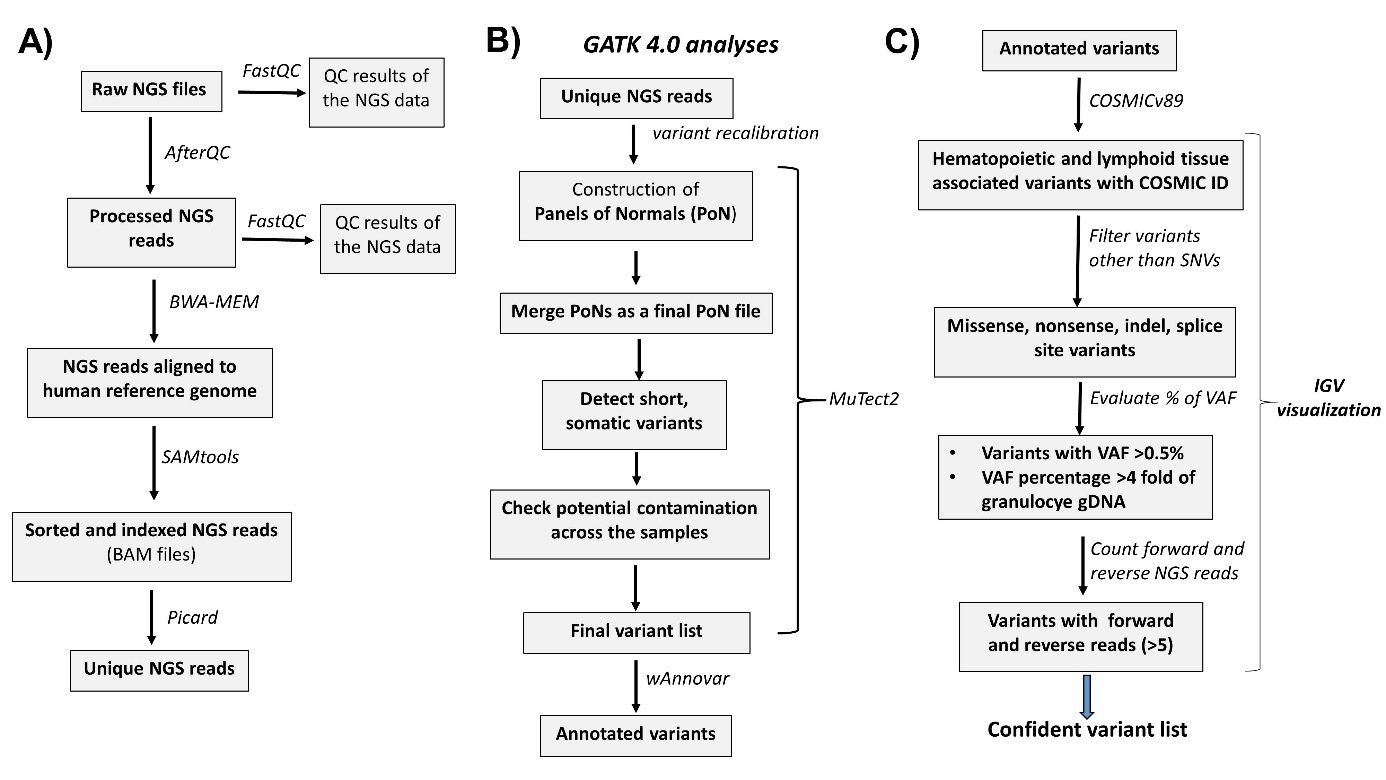


**Supplementary Figure S2. Computational bioinformatics pipeline used for identification of a confident list of somatic variants in FL cases.** The workflows showing the analysis steps until unique NGS reads (**A**), annotated variants (**B**), confident list after filtering with IGV (**C**).


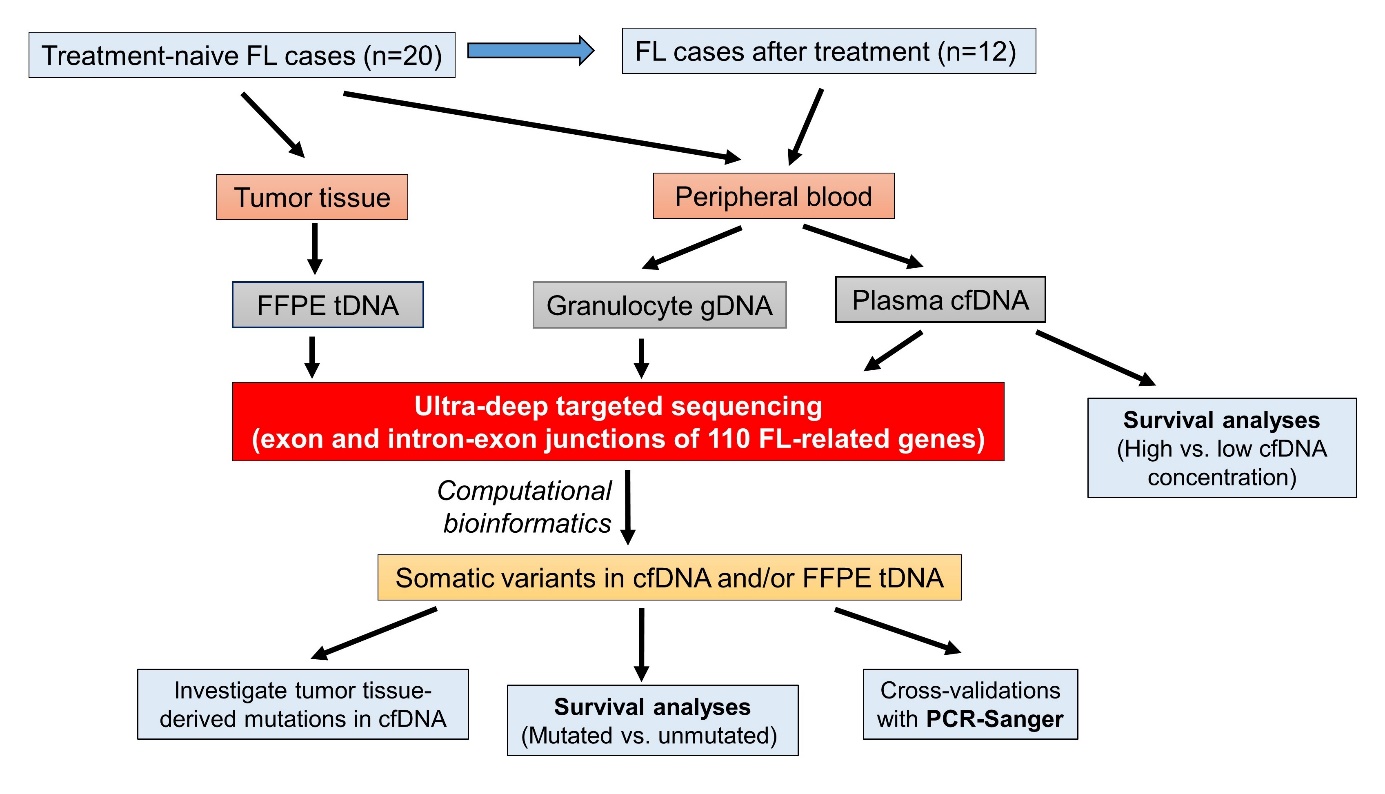


**Supplementary Figure S3. General workflow of the study.**


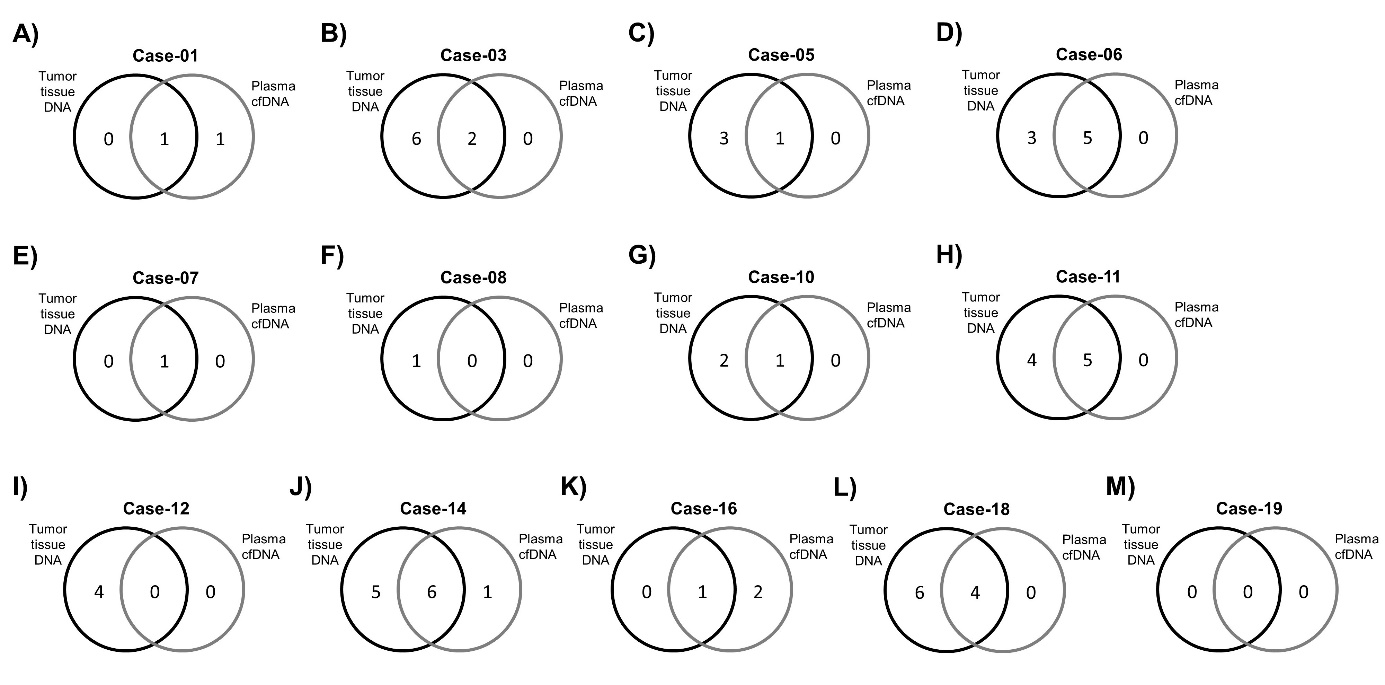


**Supplementary Figure S4. Somatic variant numbers in DNA samples of symptomatic FL cases.** The numbers of somatic missense, nonsense, indel and splice site mutations in plasma cfDNA and tumor tissue DNA for each symptomatic, treatment-naive FL patient are shown as intersecting Venn diagrams.


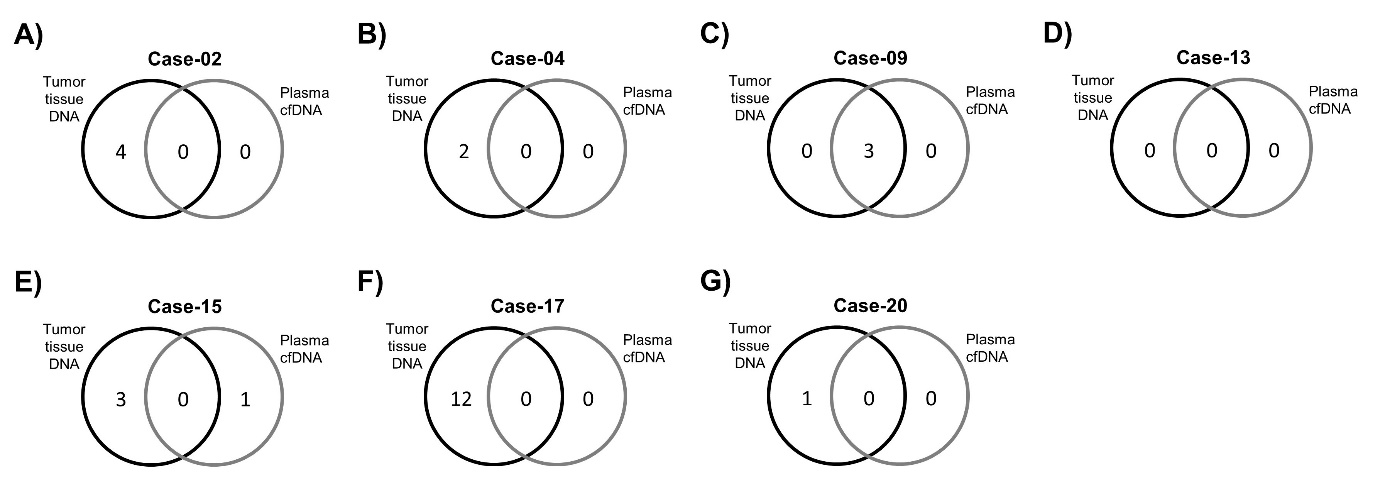


**Supplementary Figure S5. Somatic variant numbers in asymptomatic FL cases.** The number of somatic mutations (i.e. missense, nonsense, indel and splice site) for each asymptomatic FL patient are shown for cfDNA and tumor tissue DNA samples as Venn diagrams.


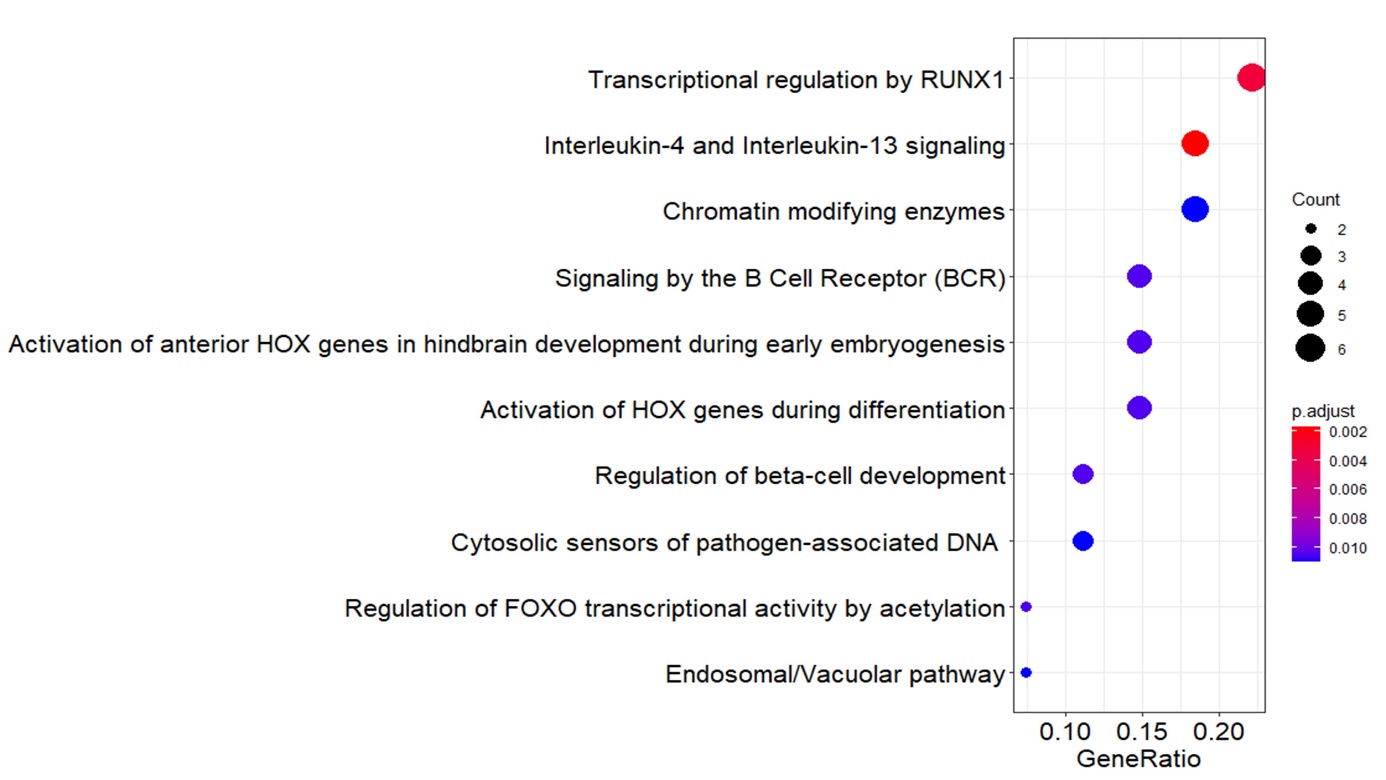


**Supplementary Figure S6. Pathways associated with the mutated genes in follicular lymphoma cases.** Thirty-one genes identified by ultra-deep next generation sequencing to be mutated in follicular lymphoma cases were used as input for pathway enrichment analyses. Pathway enrichment analysis was performed by applying the R package called “ReactomePA”, which is based on the REACTOME pathway database.


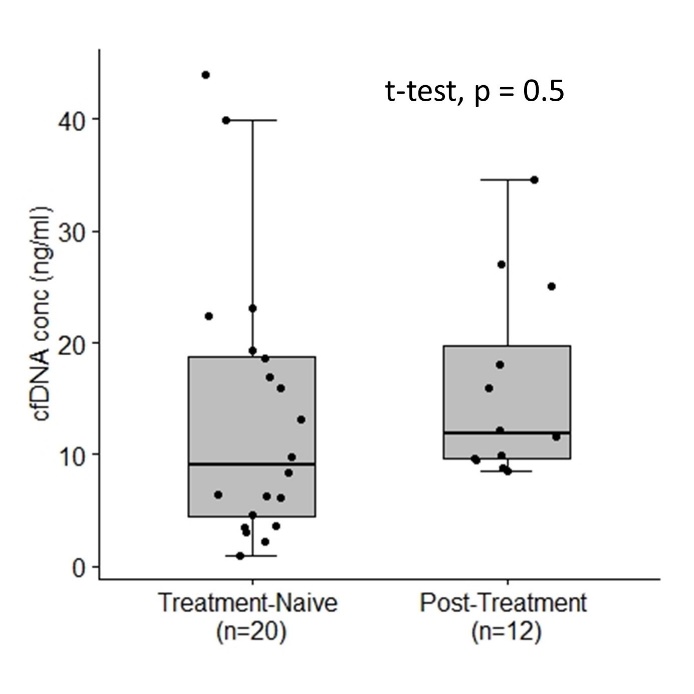


**Supplementary Figure S7. Comparison of cfDNA concentrations between treatment-naive and post-treatment FL cases.** Box-whisker plot comparing plasma cfDNA concentrations of treatment-naive and post-treatment FL patients.


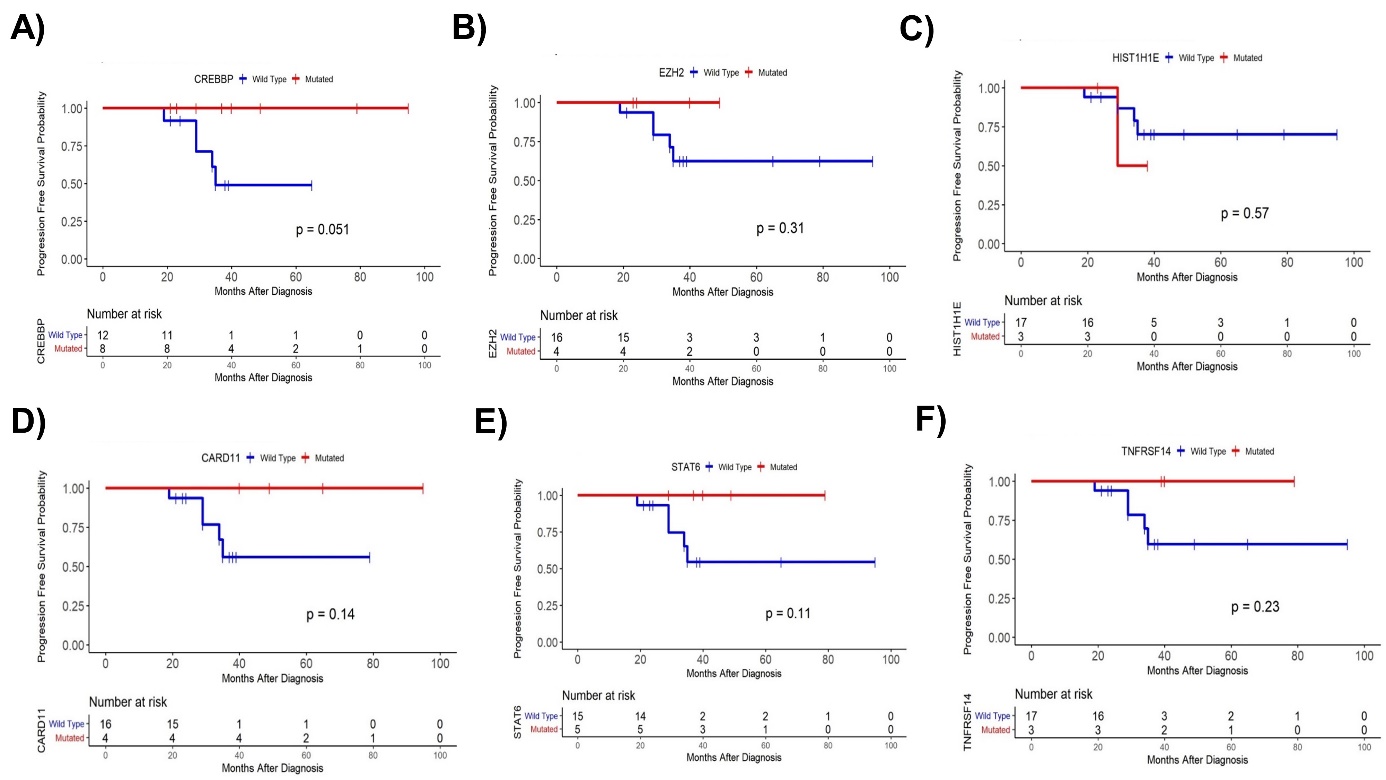


**Supplementary Figure S8. Survival curves of recurrently mutated genes not associated with FL survival.** Progression free survival curves for *CREBBP* (**A**), *EZH2* (**B**), *HIST1H1E* (**C**), *CARD11* (**D**), *STAT6* (**E**), *TNFRSF14* (**F**), which are mutated in 15% or more FL cases, are shown as Kaplan-Meier plots. p<0.05 is considered statistically significant.


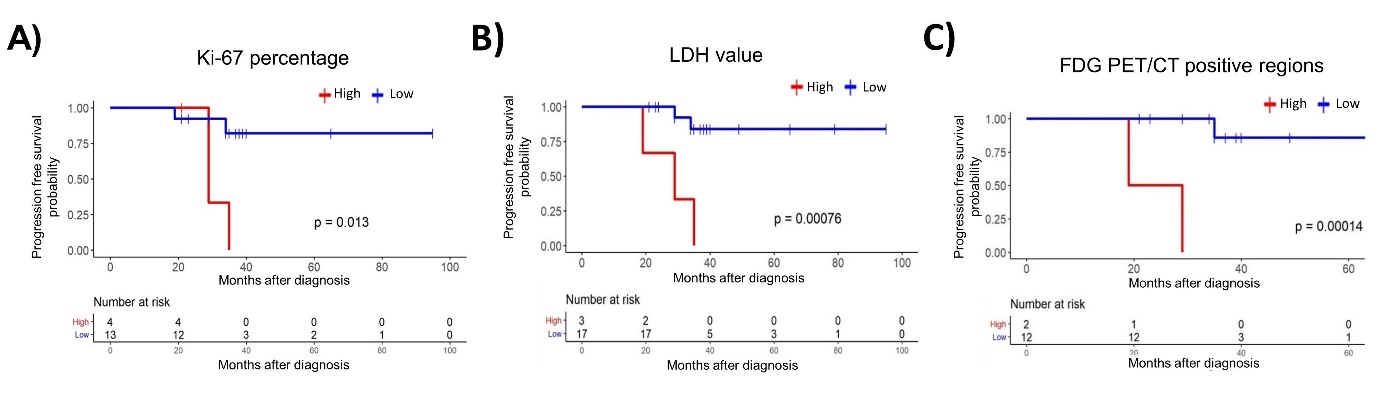


**Supplementary Figure S9. Ki-67 percentage, LDH value, or FDG PET/CT positive site number predict poor prognosis in follicular lymphoma cases.** Kaplan Meier plots showing progression free survival probability of FL patients stratified based on percentage of Ki-67 (**A**), serum LDH values (**B**), and FDG PET/CT positive region number (**C**).


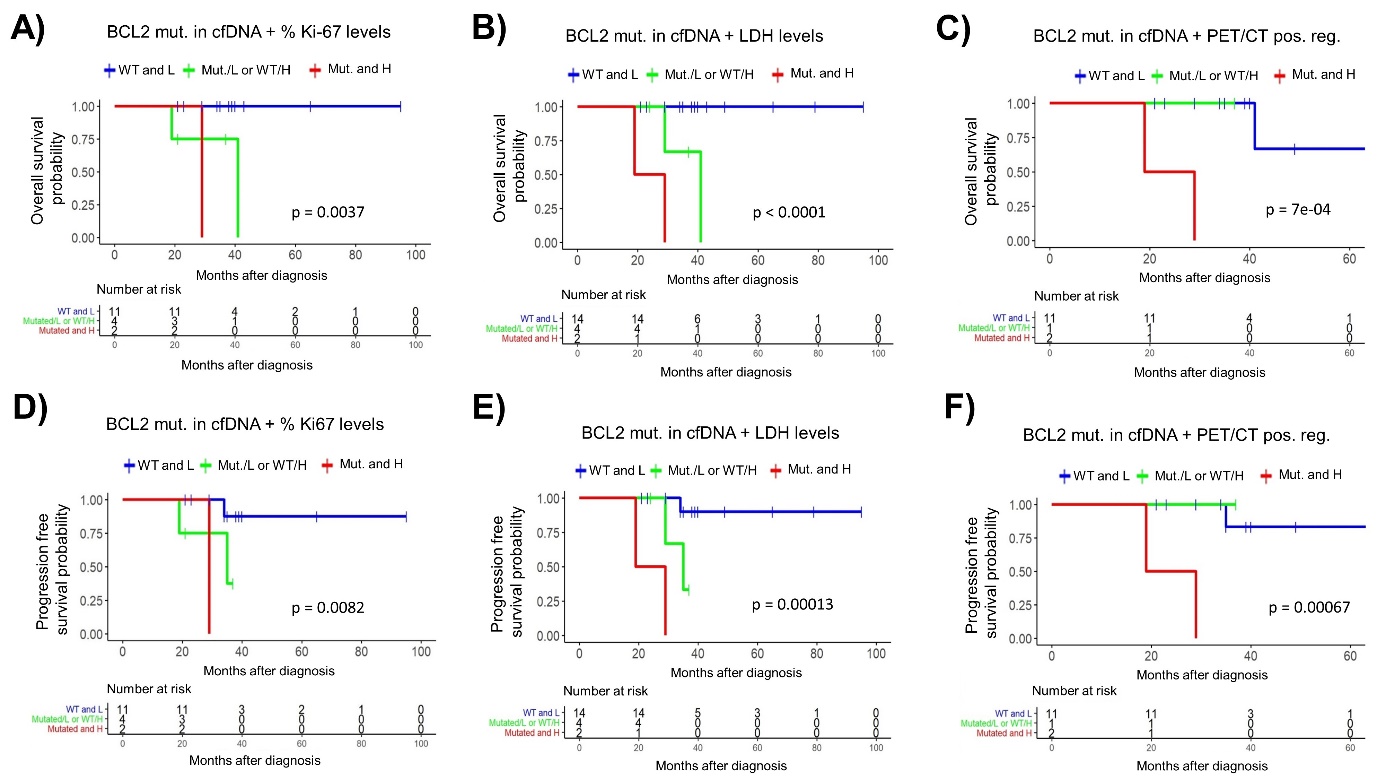


**Supplementary Figure S10. Follicular lymphoma patients with co-presence of *BCL2* cfDNA mutations and adverse clinical variables have a highly poor survival**. Kaplan-Meier plots showing the overall survival for FL patients based on the *BCL2* mutation status in cfDNA and high percentage of Ki-67 levels (**A**), high serum LDH levels (**B**), or high number of PET/CT positive regions (**C**). Kaplan-Meier plots displaying progression-free survival for FL patients with *BCL2* mutations and high percentage of Ki-67 positive cells (**D**), high LDH levels (**E**), or high number of PET/CT positive regions (**F**). p-values represent the statistical significance based on the log-rank test.


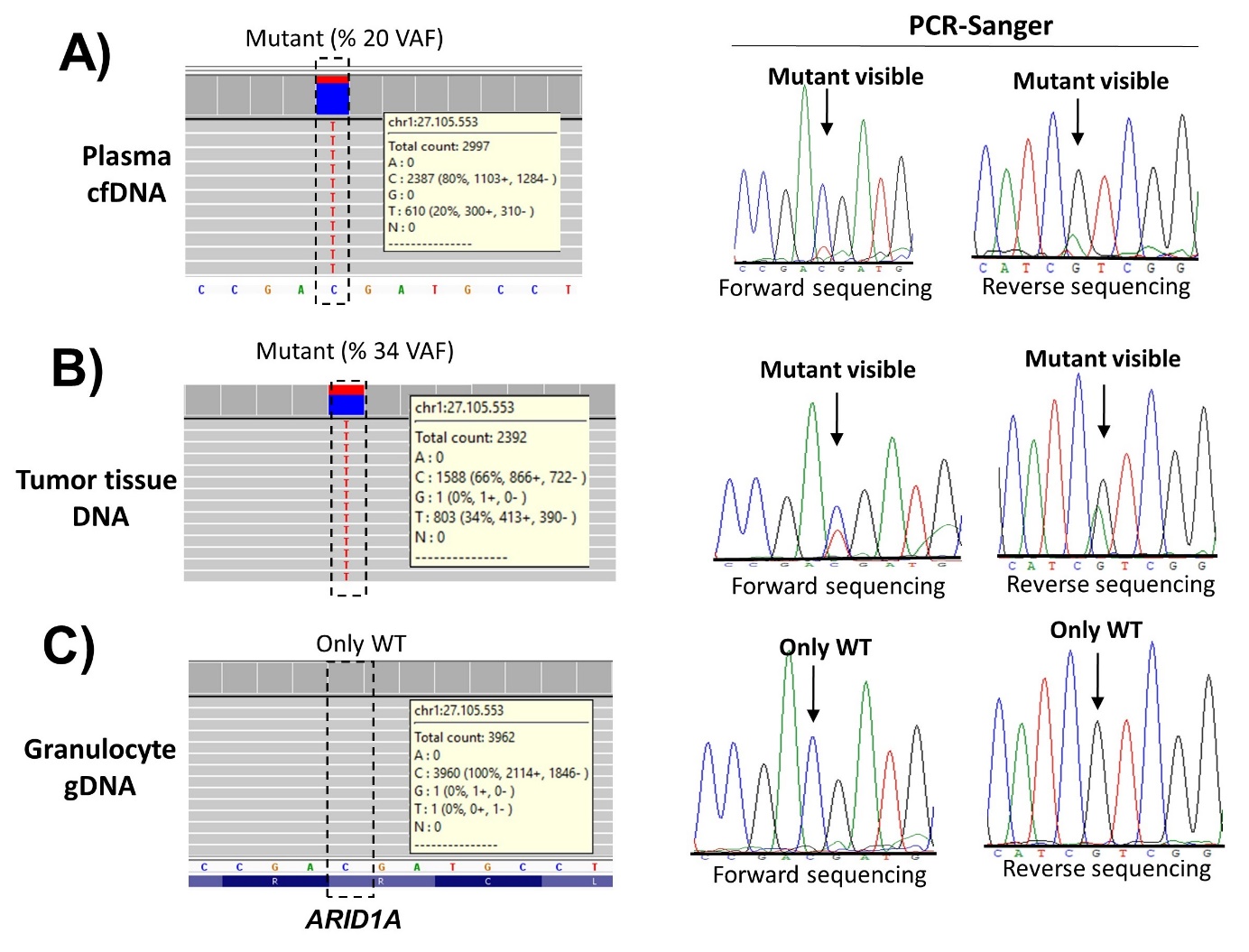


**Supplementary Figure S11. Cross-validation of the ARID1A R1722* with PCR Sanger in FL DNA samples.** ARID1A R1722* nonsense variant detected in an FL case (Case-14) through targeted ultra-deep sequencing in plasma cfDNA (**A**) and tumor tissue DNA (**B**) is shown as IGV snapshots on NGS reads (left panels) and PCR-Sanger chromatogram (right panels). The targeted and Sanger sequencing results of the patient-matched granulocyte DNA (**C**) of the same FL patient are shown as a negative control.


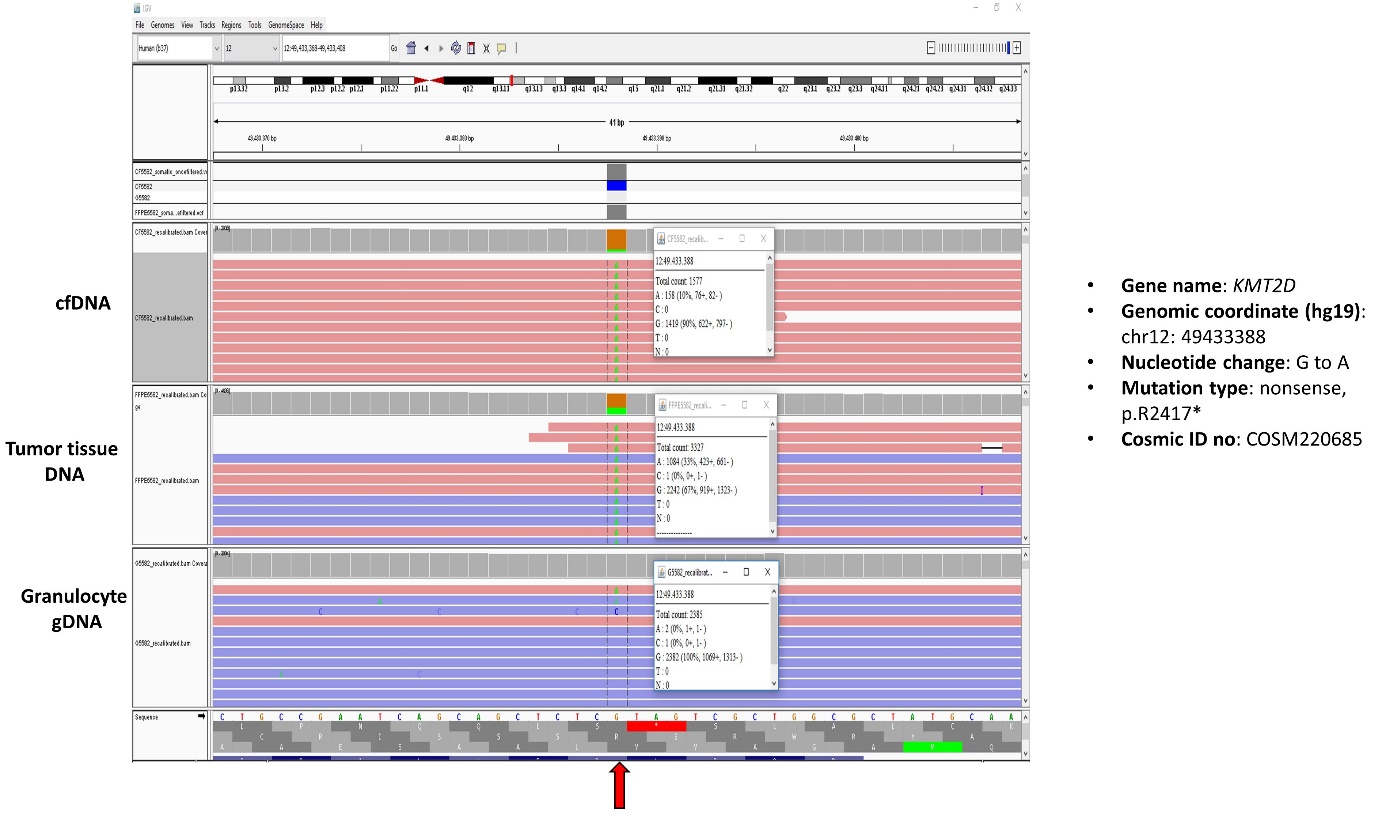


**Supplementary Figure S12. The IGV screenshot of *KMT2D* (*MLL2*) nonsense mutation detected both in cfDNA and FFPE tDNA of an FL case.** The arrow indicated the location of the variation. This mutation was previously reported in FL tumors (Pasqualucci et al. *Cell Rep*. 2014). PMID: 24388756).

**Supplementary Tables**

**Supplementary Table S1. Treatment and the timeframe of the peripheral blood sampling before and after therapy of symptomatic FL cases**

| **FL case number** | **Sampling time post-diagnosis^a^** | **Treatment type** | **Initial therapy time post-diagnosis^a^** | **Number of last chemotherapy cycle before post-therapy sampling** | **Sampling time after last chemotherapy cycle^b^** |
| --- | --- | --- | --- | --- | --- |
| **Case-01** | 54 weeks | R-CHOP | 54 weeks | 6th cycle | 64 weeks |
| **Case-03** | 8 weeks | R-CVP | 8 weeks | 6th cycle | 4 weeks |
| **Case-05** | 4 weeks | R-CHOP | 4 weeks | 5th cycle | 19 weeks |
| **Case-06** | 3 weeks | R-CVP-Radiotherapy | 4 weeks | 6th cycle | 18 weeks |
| **Case-07** | 8 weeks | R-CHOP | 13 weeks | 6th cycle | 2 weeks |
| **Case-08** | 3 weeks | R-CHOP | 3 weeks | 6th cycle | 3 weeks |
| **Case-10** | 2 weeks | R-Bendamustine | 7 weeks | 6th cycle | out of follow-up |
| **Case-11** | 8 weeks | R-Bendamustine | 8 weeks | 6th cycle | 5 weeks |
| **Case-12** | 5 weeks | R-CHOP | 5 weeks | 6th cycle | 3 weeks |
| **Case-14** | 0 week | R-CHOP | 1 week | 4th cycle | 3 weeks |
| **Case-16** | 98 weeks | R-CHOP | 99 weeks | 6th cycle | 4 weeks |
| **Case-18** | 9 weeks | R-Bendamustine | 10 weeks | 4th cycle | 6 weeks |
| **Case-19** | 6 weeks | R-CHOP | 17 weeks | 2nd cycle | 4 weeks |
| ^a^ weeks after diagnosis | |  |  |  |  |
| ^b^ weeks after last chemotherapy cycle | | |  |  |  |
| R-Bendamustine: Rituximab - Bendamustine | | |  |  |  |
| R-CHOP: Rituximab - Cyclophosphamide, Hydroxydaunomycin, Oncovin, Prednisone | | | | | |
| R-CVP: Rituximab - Cyclophosphamide, Vincristine Sulfate, Prednisone | | | | |  |

**Supplementary Table S2. List of primers and their sequences used in PCR-Sanger validation**

| **Primer Name** | **Forward Sequence** | **Reverse Sequence** |
| --- | --- | --- |
| CREBBP-C1237Y | 5'- AAATGACAGGACGGTACTTACG -3' | 5'- GGACCAGTTCACCCAAGTATG -3' |
| HIST1H1E-P131S | 5'- GGGAAGCCAAGCCTAAG -3' | 5'- GGCTTCTTCGCCTTCTTT -3' |
| ARID1A-R1722 | 5'- AATTCTGTTCTTAGGCCACTTT -3' | 5'- CACCCACCTCATACTCCTTTA -3' |
| STAT6-D523V | 5'- TATGGCTGCTCAGACTACC -3' | 5'- CCCTAGGAGATCCTGCTG -3' |
| CTSS-Y132D | 5'- ACTAAGCATTTAAAGAGCTCTACCT -3' | 5'- TTGCCTGATTCTGTGGACTG -3' |
| HIST1H1E-A65P | 5'- GCTCATTACTAAAGCTGTTGCC -3' | 5'- TGTTGTTCTTCTCCACGTCAT -3' |
| ATP6AP1-G363R | 5'- CCTCGAAGTCCACAGCAAT -3' | 5'- ACGAGGAGACTACCCTTCTT -3' |
| IRF8-Y23H | 5'- TCTGTCTTTCCAAGGATGTGTG -3' | 5'- CAGCGTGTTTCCAAGGGAT -3' |
| KMT2D-R2417 | 5'- TGCAGCTGTTTCCTTCTCC -3' | 5'- CCCTATATCGCTCCTGTCTCT -3' |

**Supplementary Table S3. Basic statistics of targeted next-generation sequencing**

| **Case number** | **Sample type** | **Raw reads** | **Raw data (G)** | **Effective (%)** | **Error (%)** | **Q20 (%)** | **Q30 (%)** | **GC (%)** |
| --- | --- | --- | --- | --- | --- | --- | --- | --- |
| Case-01 | cfDNA | 26542159 | 7.96 | 92.33 | 0.01 | 97.06 | 93.09 | 47.97 |
| Case-02 | cfDNA | 29995498 | 9 | 93.71 | 0.01 | 97.23 | 93.38 | 47.5 |
| Case-03 | cfDNA | 32619874 | 9.79 | 96.05 | 0.01 | 97.41 | 93.69 | 46.63 |
| Case-04 | cfDNA | 28189581 | 8.46 | 95.28 | 0.01 | 97.23 | 93.38 | 47.84 |
| Case-05 | cfDNA | 33659244 | 10.1 | 92.9 | 0.01 | 97.16 | 93.25 | 47.48 |
| Case-06 | cfDNA | 42135475 | 12.64 | 88.61 | 0.01 | 97.42 | 93.72 | 48.16 |
| Case-07 | cfDNA | 62746738 | 18.8 | 86.69 | 0.01 | 96.86 | 92.67 | 52.93 |
| Case-08 | cfDNA | 58496258 | 17.5 | 91.55 | 0.01 | 97.14 | 93.25 | 53.14 |
| Case-09 | cfDNA | 21410620 | 6.4 | 92.24 | 0.01 | 96.88 | 92.6 | 47.23 |
| Case-10 | cfDNA | 31747997 | 9.5 | 96.09 | 0.01 | 97.42 | 93.75 | 47.09 |
| Case-11 | cfDNA | 37119371 | 11.1 | 89.09 | 0.01 | 97 | 93 | 51.03 |
| Case-12 | cfDNA | 31856623 | 9.6 | 95.09 | 0.01 | 97.5 | 93.89 | 46.84 |
| Case-13 | cfDNA | 24846277 | 7.5 | 94.59 | 0.01 | 96.06 | 91.01 | 46.3 |
| Case-14 | cfDNA | 24546828 | 7.4 | 96.56 | 0.01 | 97.56 | 94.17 | 46.68 |
| Case-15 | cfDNA | 30472773 | 9.1 | 96.41 | 0.01 | 96.53 | 91.83 | 50.8 |
| Case-16 | cfDNA | 37679176 | 11.3 | 87.73 | 0.01 | 97.49 | 93.93 | 50.47 |
| Case-17 | cfDNA | 34193278 | 10.3 | 97.21 | 0.01 | 96.31 | 91.38 | 51.72 |
| Case-18 | cfDNA | 26756794 | 8 | 89.97 | 0.02 | 96.33 | 91.18 | 46.87 |
| Case-19 | cfDNA | 27017054 | 8.1 | 93.6 | 0.02 | 96.16 | 90.84 | 46.6 |
| Case-20 | cfDNA | 27980837 | 8.4 | 92.94 | 0.02 | 96.21 | 90.97 | 46.78 |
| Case-01 | cfDNA-AT | 32620105 | 9.8 | 87.29 | 0.01 | 97.38 | 93.77 | 47.5 |
| Case-03 | cfDNA-AT | 34349554 | 10.3 | 94.42 | 0.01 | 97.44 | 93.76 | 46.8 |
| Case-05 | cfDNA-AT | 29994313 | 9 | 97.5 | 0.01 | 97.66 | 94.34 | 46.47 |
| Case-06 | cfDNA-AT | 23600055 | 7.1 | 93.95 | 0.01 | 96.52 | 92.01 | 47.19 |
| Case-07 | cfDNA-AT | 24112384 | 7.2 | 98.05 | 0.01 | 97.46 | 93.94 | 47.17 |
| Case-08 | cfDNA-AT | 32133893 | 9.6 | 97.92 | 0.01 | 97.12 | 93.17 | 46.97 |
| Case-11 | cfDNA-AT | 32141097 | 9.6 | 96.2 | 0.01 | 96.15 | 91.05 | 53.13 |
| Case-12 | cfDNA-AT | 35346954 | 10.6 | 96.50 | 0.01 | 97.07 | 93.07 | 51.60 |
| Case-14 | cfDNA-AT | 31354302 | 9.4 | 95.56 | 0.01 | 96.89 | 92.72 | 50.46 |
| Case-16 | cfDNA-AT | 28475159 | 8.5 | 98.32 | 0.01 | 96.91 | 92.68 | 48.2 |
| Case-18 | cfDNA-AT | 27387129 | 8.2 | 93.53 | 0.01 | 98.28 | 95.53 | 50.58 |
| Case-19 | cfDNA-AT | 33653084 | 10.1 | 92.77 | 0.01 | 98.37 | 95.97 | 49.86 |
| Case-01 | FFPE tDNA | 33009228 | 9.9 | 93.5 | 0.01 | 96.99 | 92.78 | 51.27 |
| Case-02 | FFPE tDNA | 21727482 | 6.52 | 86.79 | 0.01 | 95.44 | 90.22 | 48.91 |
| Case-03 | FFPE tDNA | 32671752 | 9.8 | 95.98 | 0.01 | 96.66 | 92.32 | 46.73 |
| Case-04 | FFPE tDNA | 64090823 | 19.23 | 95.76 | 0.01 | 96.97 | 92.73 | 52.3 |
| Case-05 | FFPE tDNA | 34065445 | 10.22 | 95.71 | 0.01 | 96.95 | 92.77 | 48.19 |
| Case-06 | FFPE tDNA | 34735008 | 10.42 | 90.87 | 0.01 | 96.16 | 91.52 | 48.25 |
| Case-07 | FFPE tDNA | 22383054 | 6.7 | 95.55 | 0.01 | 97.12 | 93.21 | 48.65 |
| Case-08 | FFPE tDNA | 22554851 | 6.8 | 97.46 | 0.01 | 96.05 | 90.9 | 47.12 |
| Case-09 | FFPE tDNA | 34789809 | 10.4 | 96.85 | 0.01 | 97.16 | 93.32 | 46.34 |
| Case-10 | FFPE tDNA | 33694729 | 10.1 | 98.39 | 0.01 | 97.01 | 93.06 | 46.09 |
| Case-11 | FFPE tDNA | 25135696 | 7.5 | 95.61 | 0.01 | 97.2 | 93.41 | 46.84 |
| Case-12 | FFPE tDNA | 25587131 | 7.7 | 96.79 | 0.01 | 96.28 | 91.32 | 49.36 |
| Case-13 | FFPE tDNA | 25262116 | 7.6 | 95.51 | 0.01 | 97.35 | 93.73 | 47.23 |
| Case-14 | FFPE tDNA | 29828087 | 8.9 | 98.85 | 0.01 | 96.17 | 91.19 | 44.95 |
| Case-15 | FFPE tDNA | 58048036 | 17.4 | 88.74 | 0.01 | 96.16 | 91.14 | 49.21 |
| Case-16 | FFPE tDNA | 102120822 | 30.6 | 83.40 | 0.01 | 96.17 | 91.16 | 47.59 |
| Case-17 | FFPE tDNA | 25348580 | 7.6 | 48.93 | 0.01 | 96.03 | 91.12 | 53.75 |
| Case-18 | FFPE tDNA | 37352964 | 11.2 | 64.16 | 0.02 | 95.42 | 90.09 | 49.52 |
| Case-19 | FFPE tDNA | 25639445 | 7.7 | 79.21 | 0.02 | 95.24 | 89.64 | 43.89 |
| Case-20 | FFPE tDNA | 22813618 | 6.8 | 82.98 | 0.02 | 94.94 | 89.1 | 45.67 |
| Case-01 | gDNA | 21882109 | 6.56 | 98.58 | 0.01 | 97.12 | 93 | 47.99 |
| Case-02 | gDNA | 31057521 | 9.3 | 98.36 | 0.01 | 96.6 | 92.05 | 48.51 |
| Case-03 | gDNA | 21557107 | 6.47 | 98.77 | 0.01 | 96.97 | 92.69 | 47.71 |
| Case-04 | gDNA | 21347311 | 6.4 | 98.99 | 0.01 | 97.03 | 92.76 | 47.77 |
| Case-05 | gDNA | 17999553 | 5.4 | 98.35 | 0.01 | 97.06 | 92.84 | 48.14 |
| Case-06 | gDNA | 22065195 | 6.62 | 98.84 | 0.01 | 97.01 | 92.78 | 47.94 |
| Case-07 | gDNA | 21827691 | 6.5 | 99.52 | 0.01 | 96.96 | 92.59 | 47.73 |
| Case-08 | gDNA | 24348793 | 7.3 | 99.42 | 0.01 | 97.18 | 93.09 | 47.36 |
| Case-09 | gDNA | 28307102 | 8.5 | 99.3 | 0.01 | 97.13 | 93.07 | 47.45 |
| Case-10 | gDNA | 28796310 | 8.6 | 99.4 | 0.01 | 97.13 | 93.09 | 48.34 |
| Case-11 | gDNA | 27052238 | 8.1 | 99.55 | 0.01 | 96.5 | 91.59 | 47.34 |
| Case-12 | gDNA | 28036495 | 8.4 | 99.18 | 0.01 | 97.27 | 93.33 | 47.94 |
| Case-13 | gDNA | 28620332 | 8.6 | 99.36 | 0.01 | 97.03 | 92.88 | 47.94 |
| Case-14 | gDNA | 23764241 | 7.1 | 99.5 | 0.01 | 97.11 | 92.97 | 47.58 |
| Case-15 | gDNA | 32222469 | 9.7 | 98.72 | 0.01 | 96 | 90.68 | 52.48 |
| Case-16 | gDNA | 37571829 | 11.3 | 98.58 | 0.01 | 96.06 | 90.8 | 52.72 |
| Case-17 | gDNA | 38301358 | 11.5 | 98.58 | 0.01 | 95.89 | 90.43 | 53.73 |
| Case-18 | gDNA | 31245081 | 9.4 | 98.22 | 0.01 | 96.72 | 92.31 | 47.33 |
| Case-19 | gDNA | 25116904 | 7.5 | 98.07 | 0.01 | 96.56 | 91.97 | 47.31 |
| Case-20 | gDNA | 25337582 | 7.6 | 98.65 | 0.01 | 96.45 | 91.72 | 48.38 |

**FFPE tDNA**: Formalin-fixed paraffin-embedded tumor tissue DNA
**cfDNA**: Circulating cell free DNA
**gDNA**: Granulocyte genomic DNA
**cfDNA-AT**: Circulating cell free DNA after therapy

**Supplementary Table S5. Contingency tables showing the relationship between *BCL2* or *CCND3* mutations and follicular lymphoma survival**

**A) Presence of mutation in FFPE tDNA and/or cfDNA vs. overall survival.**

|  |  | ***BCL2*** |  |  |  |  | ***CCND3*** |  |
| --- | --- | --- | --- | --- | --- | --- | --- | --- |
|  | Mutated | Wild Type | Total |  |  | Mutated | Wild Type | Total |
| Survived | **3** | **13** | 16 |  | Survived | **0** | **16** | 16 |
| Not Survived | **3** | **1** | 4 |  | Not Survived | **1** | **3** | 4 |
| Total | 6 | 14 | 20 |  | Total | 1 | 19 | 20 |
|  | p-value*: | **0.06** |  |  |  | p-value: | **0.2** |  |

**B) Presence of mutation in cfDNA vs. overall survival.**

|  |  | ***BCL2*** |  |  |  |  | ***CCND3*** |  |
| --- | --- | --- | --- | --- | --- | --- | --- | --- |
|  | Mutated | Wild Type | Total |  |  | Mutated | Wild Type | Total |
| Survived | **2** | **14** | 16 |  | Survived | **0** | **16** | 16 |
| Not Survived | **3** | **1** | 4 |  | Not Survived | **0** | **4** | 4 |
| Total | 5 | 15 | 20 |  | Total | 0 | 20 | 20 |
|  | p-value: | **0.03** |  |  |  | p-value: | **1** |  |

***:** All p values are based on the Fisher’s exact test.
